# Expression profiles of proton-sensing G-protein coupled receptors in common skin tumors

**DOI:** 10.1038/s41598-020-71700-9

**Published:** 2020-09-18

**Authors:** Wybke Klatt, Susanne Wallner, Christoph Brochhausen, Judith A. Stolwijk, Stephan Schreml

**Affiliations:** 1 Department of Dermatology, University Medical Center Regensburg, Franz-Josef-Strauß-Allee 11, 93053 Regensburg, Germany; 2 Institute of Pathology, University of Regensburg, Franz-Josef-Strauß-Allee 11, 93053 Regensburg, Germany; 3 Institute of Analytical Chemistry, Chemo- and Biosensors, Faculty of Chemistry and Pharmacy, University of Regensburg, Universitätsstraße 31, 93053 Regensburg, Germany

**Keywords:** Cancer, Medical research

## Abstract

The proton-sensing GPCRs (pH-GPCRs) GPR4 (GPR19), TDAG8 (GPR65, T-cell death associated gene 8), OGR1 (GPR68, ovarian cancer GPCR1), and G2A (GPR132, G2 accumulation protein) are involved in sensing and transducing changes in extracellular pH (pH_e_). Extracellular acidification is a central hallmark of solid cancer. pH-GPCR function has been associated with cancer cell proliferation, adhesion, migration and metastasis, as well as with modulation of the immune system. Little is known about the expression levels and role of pH-GPCRs in skin cancer. To better understand the functions of pH-GPCRs in skin cancer in vivo, we examined the expression-profiles of GPR4, TDAG8, OGR1 and G2A in four common skin tumors, i.e. squamous cell carcinoma (SCC), malignant melanoma (MM), compound nevus cell nevi (NCN), basal cell carcinoma (BCC). We performed immunohistochemistry and immunofluorescence staining on paraffin-embedded tissue samples acquired from patients suffering from SCC, MM, NCN or BCC. We show the expression of pH-GPCRs in four common skin cancers. Different expression patterns in the investigated skin cancer types indicate that the different pH-GPCRs may have distinct functions in tumor progression and serve as novel therapeutic targets.

## Introduction

In 2019, the United States are projecting 1,762,450 new cancer cases to occur^1^. Over the past decade particularly skin cancer, one of the most common types of malignancies, has shown an increasing incidence^2,3^. Among non-melanoma skin cancers, squamous cell carcinoma (SCC) grows slow over months and only 4% of the SCC tumours metastasise, leading to significant patient morbidity^4^. SCC is the second most-frequent cutaneous malignancy, preceded in frequency by the basal cell carcinoma (BCC). BCC is characterised by a slow growing behaviour and metastases are extremely rare^5^. Nevus cell nevi (NCN) are benign melanocytic lesions which do not require any intervention^6^. The malignant melanoma (MM) develops from the nevus cell nevus in one third of the cases^7^. Although MM represents only 2% of the malignant skin cancer incidents, it is still one of the deadliest skin cancers with a rapid systematic dissemination^8,9^.

G-protein coupled receptors (GPCRs) are one of the most diverse classes of cell surface receptors with over 500 representatives in eukaryotes, including animals, plants, fungi and the human body, where they fulfil a multitude of crucial individual tasks^10,11^.

The pH-sensitive GPCRs (pH-GPCRs) GPR4 (GPR19), TDAG8 (GPR65, T-cell death associated gene 8), OGR1 (GPR68, ovarian cancer GPCR1) and G2A (GPR132, G2 accumulation protein) are activated by protons in the extracellular environment, presumably through binding to specific histidine residues at the extracellular surface of these receptors^12^. Under healthy conditions the central function of these proton sensors seems the maintenance of homeostasis. However, in tumours it is believed that certain pH-GPCRs may help to establish a growth advantage while others inhibit growth^13,14^. Moreover, there are differences between the tumor types^15^.

Tumor pH often differs from normal tissue. While standard stromal cells maintain intracellular pH (pH_i_) in a narrow range of 6.9–7.2 compared to the extracellular pH (pH_e_) of 7.2–7.4, tumors exhibit lower pH_e_ (6.2–7.0) and preserve an alkaline intracellular pH (7.2–7.7)^15,16^. This pH dysregulation, termed reversed (= inside-out) pH gradient (pH_e_ < pH_i_), has been recognized as a hallmark of cancer^13,17,18^. The lower pH_e_ is typically caused by disorganised vascularisation, specific proton transporters and metabolic changes. Insufficient vascularisation leads to the development of hypoxic regions, where instead of aerobic glucose metabolism anaerobic glycolysis takes over, producing lactate^19,20^. In addition, different transporters/pumps are involved in the regulation of tumor pH_i_ and pH_e_, including monocarboxylate transporters 1–4 (MCT), the Na^+^/H^+^ exchanger 1 (NHE1), HCO_3_^−^ transporters (NBCs), vacuolar ATPases (V-ATPase) as well as different carboanhydrases (CAII, CAIV, CAXII)^12,17–19^. Adaption of cancer cells to extracellular acidosis drives tumor progression by affecting cell turnover, promoting metastasis and metabolic changes^14,17–19,21^.

pH-GPCR function has been associated with cancer cell proliferation, adhesion, migration and metastasis, as well as with modulation of the immune system^12,14,22–30^, but so far there is no precise concept that links individual pH-GPCR expression to certain cancer cell function.

Only little is known on the expression of proton-sensing GPCRs GPR4, TDAG8, OGR1 and G2A in the skin and especially in skin cancer^15^. The pH-GPCRS GPR4, TDAG8, OGR1, and G2A appear to be expressed in the skin, but data on protein level that clearly link expression to specific skin cells are sparse^14^. Nassios et al.^15^ provided first evidence of pH-GPCR expression on protein level in selected rare skin cancers merkel cell carcinoma, dermatofibrosarcoma protuberans, atypical fibroxanthoma and pleomorphic dermal sarcoma.

In this study, we have focussed on analysing the expression levels of the respective pH-GPCRs in tissue samples of four of the most common skin cancer types, SCC, MM, NCN and BCC. The identification of characteristic expression patterns of the four different pH GPCRs in the respective tumour types may help to contribute to a better individual therapy of the four tumour types and enable a more substantial insight into considering pH-GPCRs as therapeutic target.

## Results

We summarised our findings of immunohistochemistry data for individual SCC, MM, NCN and BCC tumors from each 5–6 patients. In order to present more data, we performed additional immunohistochemistry on TMA-format including 24–27 samples per tumor type. Figures 1, 2, 3 and 4 show representative IHC and IF staining results on tissue samples with SCC, MM, NCN and BCC for GPR4, TDAG8, OGR1 and G2A. Images from IHC/IF on all other samples can be found in the Supplementary Figures S1–S17. Staining results for MM and NCN were divided in epidermal and dermal portions. Figures 5a–d and 6a–d summarize both, regular IHC and TMA results. General patient information is given in Supplementary Table S1. Additional TMA data and scores are shown in Supplementary Figure S18 and Supplementary Tables S2–S5. In the following the combined immunohistochemistry and immunofluorescence data as well as the supporting TMA data are discussed for each pH-GPCR.

**Figure 1:**
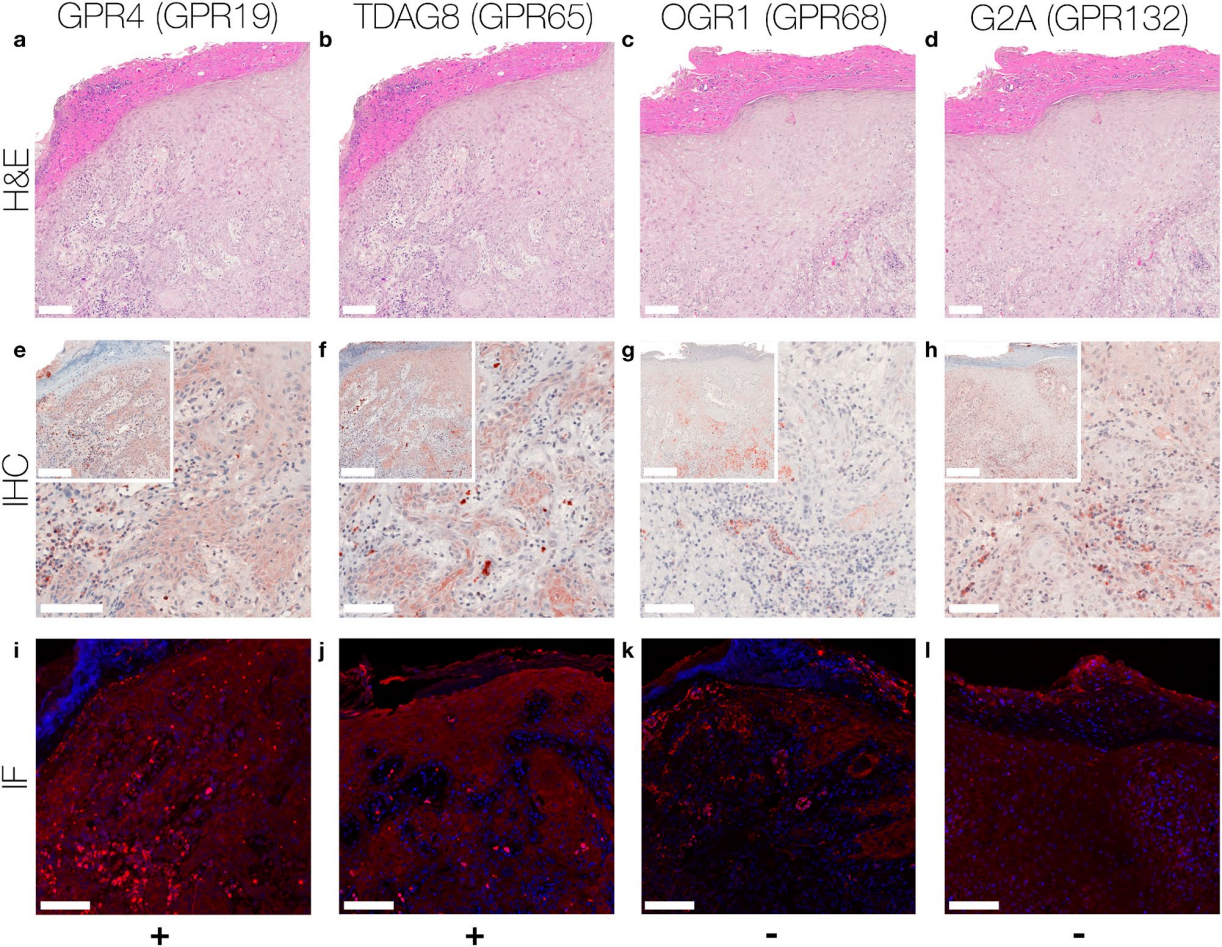
Immunohistochemistry and Immunofluorescence of SCC. Immunohistochemical and immunofluorescent staining for GPR4 (GPR19), TDAG8 (GPR65), OGR1 (GPR68) and G2A (GPR132) in SCC tissue. (**a**–**d**) histochemical H&E staining, (**e**–**h**) immunohistochemical staining, inserted images present a 2 × larger field of view, (**i**–**l**) immunofluorescence staining, red: secondary antibody label, blue: DAPI. Scale bars correspond to 100 μm (**a**–**l**: patient 1). Scores (bottom row) were assigned for ++: strong positive/positive reactions; +: weak positive/partial positive reaction; −: negative reaction. This SCC shows no expression of OGR1 and G2A in tumor cells, only several peritumoral lymphocytes appeared to be positive. The expression of GPR4 and TDAG8 is partial positive. For additional stainings of other SCC, see Figures S1–S4.

**Figure 2:**
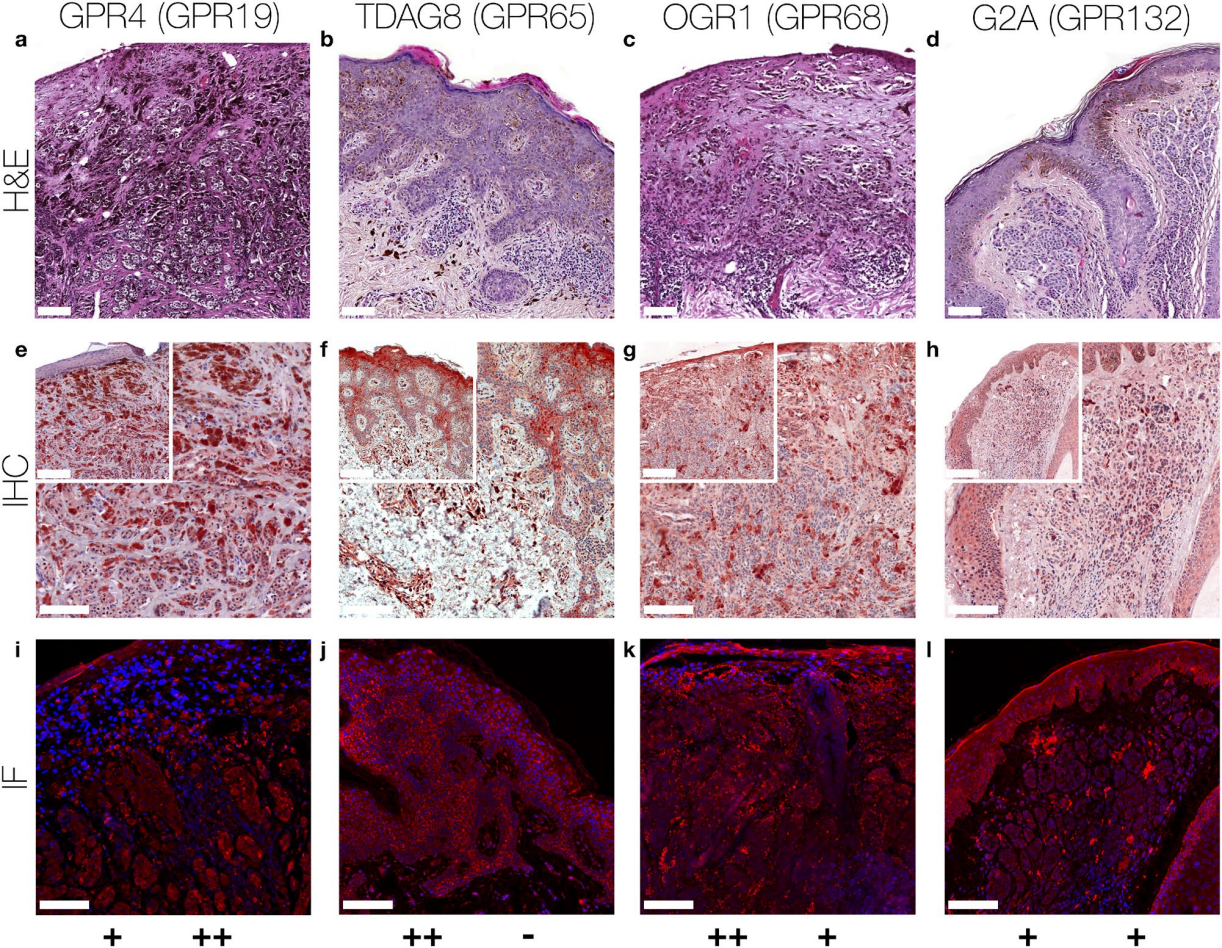
Immunohistochemistry and Immunofluorescence of MM. Immunohistochemical and immunofluorescent staining for GPR4 (GPR19), TDAG8 (GPR65), OGR1 (GPR68) and G2A (GPR132) in MM tissue. (**a**–**d**) histochemical H&E staining, (**e**–**h**) immunohistochemical staining, inserted images present a 2 × larger field of view, (**i**–**l**) immunofluorescence staining, red: secondary antibody label, blue: DAPI. Scale bars correspond to 100 μm (**a**,**c**,**e**,**g**,**i**,**k** patient 8; **b**,**d**,**f**,**h**,**j**,**l** patient 9). Scores (bottom row) were assigned for ++: strong positive/positive reactions; +: weak positive/partial positive reaction; −: negative reaction for the epidermal (left score) and the dermal (right score) portion. The MM shows a strong positive expression of TDAG8 and OGR1 in the epidermal portions. The epidermal expression of GPR4 and G2A is partial positive. There is a strong positive dermal expression of GPR4. OGR1 and G2A are weak positive regarding the dermal area and TDAG8 is not expressed dermally. Generally, smaller tumor cells within the tumor appear to be weakly positive, while below the epidermis multinuclear giant tumor cells with altered nucleus-cytoplasmic ratio are strongly expressed. For additional stainings of other MM, see Figures S5–S8.

**Figure 3:**
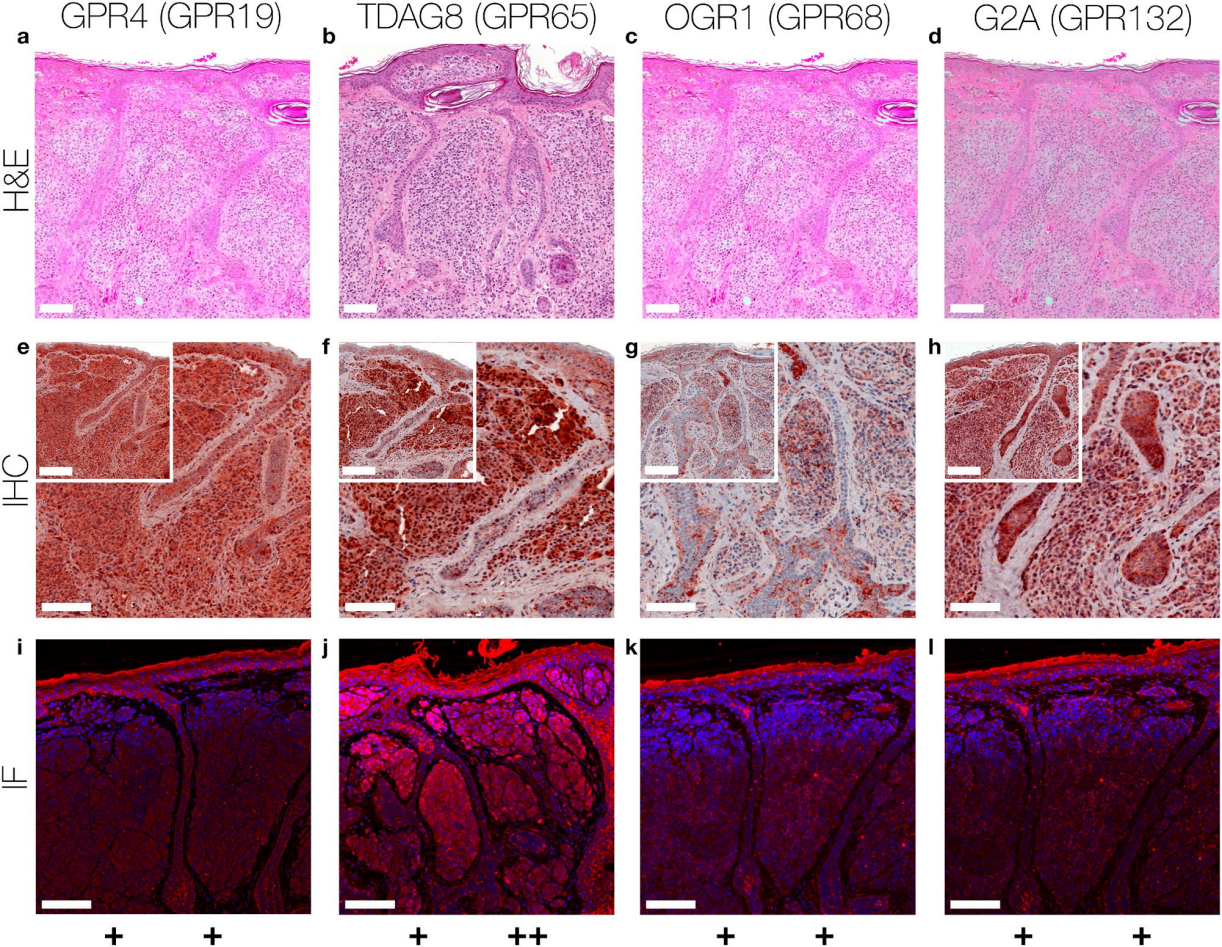
Immunohistochemistry and Immunofluorescence of NCN. Immunohistochemical and immunofluorescent staining for GPR4 (GPR19), TDAG8 (GPR65), OGR1 (GPR68) and G2A (GPR132) in NCN tissue. (**a**–**d**) histochemical H&E staining, (**e**–**h**) immunohistochemical staining, inserted images present a 2 × larger field of view, (**i**–**l**) immunofluorescence staining, red: secondary antibody label, blue: DAPI. Scale bars correspond to 100 μm (**a**–**l** patient 15). Scores (bottom row) were assigned for ++: strong positive/positive reactions; +: weak positive/partial positive reaction; −: negative reaction for the epidermal (left score) and the dermal (right score) portion. The NCN shows a partial positive expression of all GPCRs on the epidermal portions. There are even some epidermal tumor cells strongly expressed in the TDAG8. Regarding the dermal area GPR4, OGR1 and G2A are weak positive, while MM shows a significantly increased expression of TDAG8. For additional stainings of other NCN, see Figures S9–S12.

**Figure 4:**
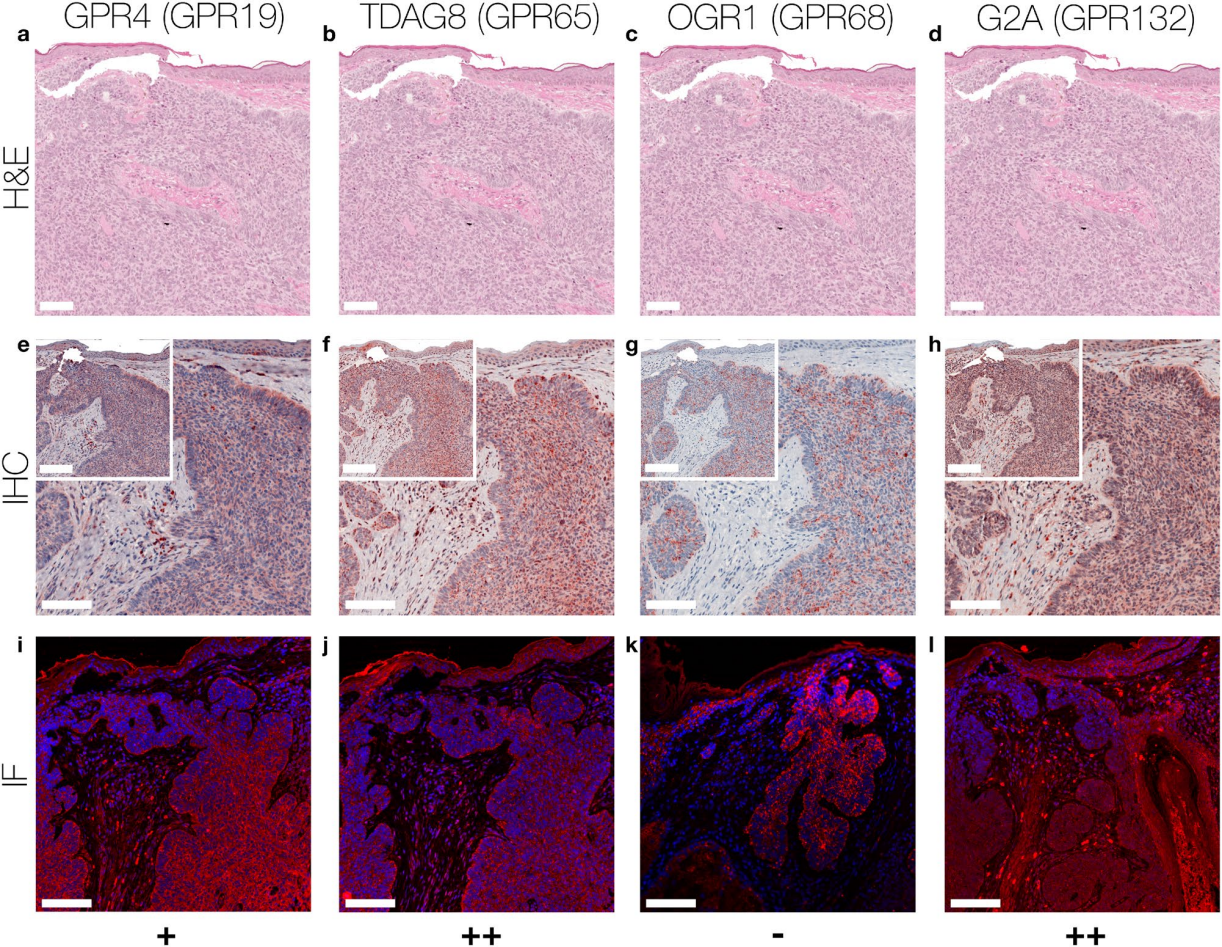
Immunohistochemistry and Immunofluorescence of BCC. Immunohistochemical and immunofluorescent staining for GPR4 (GPR19), TDAG8 (GPR65), OGR1 (GPR68) and G2A (GPR132) in BCC tissue. (**a**–**d**) histochemical H&E staining, (**e**–**h**) immunohistochemical staining, inserted images present a 2 × larger field of view, (**i**–**l**) immunofluorescence staining, red: secondary antibody label, blue: DAPI. Scale bars correspond to 100 μm (**a**–**l** patient 20). Scores (bottom row) were assigned for ++: strong positive/positive reactions; +: weak positive/partial positive reaction; −: negative reaction. The expression of TDAG8 and G2A on the surface of BCC tumor cells is significantly increased, while the expression of GPR4 is weakly positive. No expression of OGR1 is detected. For additional stainings of other BCC, see Figures S13–S17.

**Figure 5:**
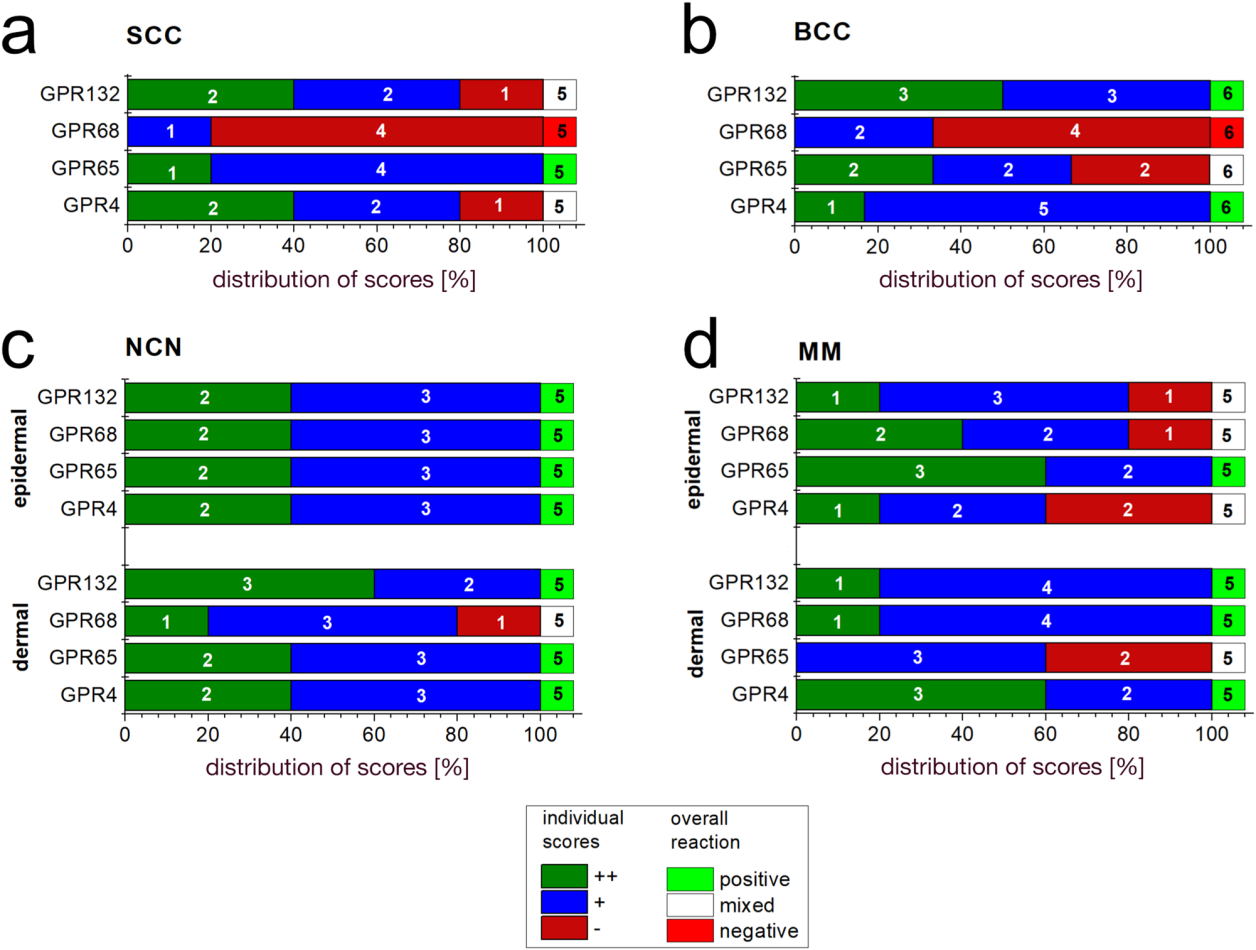
IHC-Score results for different pH-GPCRs in common skin tumors. Fractional distribution of the scores (green bar, ++: strong positive/positive reaction; +, blue bar: weak positive/ partial positive reaction; −, red bar: negative reaction) for GPR4 (GPR19), TDAG (GPR65), OGR1 (GPR68) and G2A (GPR132) of immunohistochemically stained skin tumors (**a**) SCC, (**b**) BCC, (**c**) NCN, (**d**) MM. MM and NCN are further subclassified in epidermal and dermal portions. Numbers in bars indicate the occurrence of a respective score. The sum of all scores is 100%. Overall evaluation is indicated by a green, white or red box (beyond 100% scale): green box: overall positive reactions; red box: overall negative reactions; white box: mixed reactions without clear majority in favour of one specific reaction. Numbers in this box indicate the total number of samples investigated.

**Figure 6:**
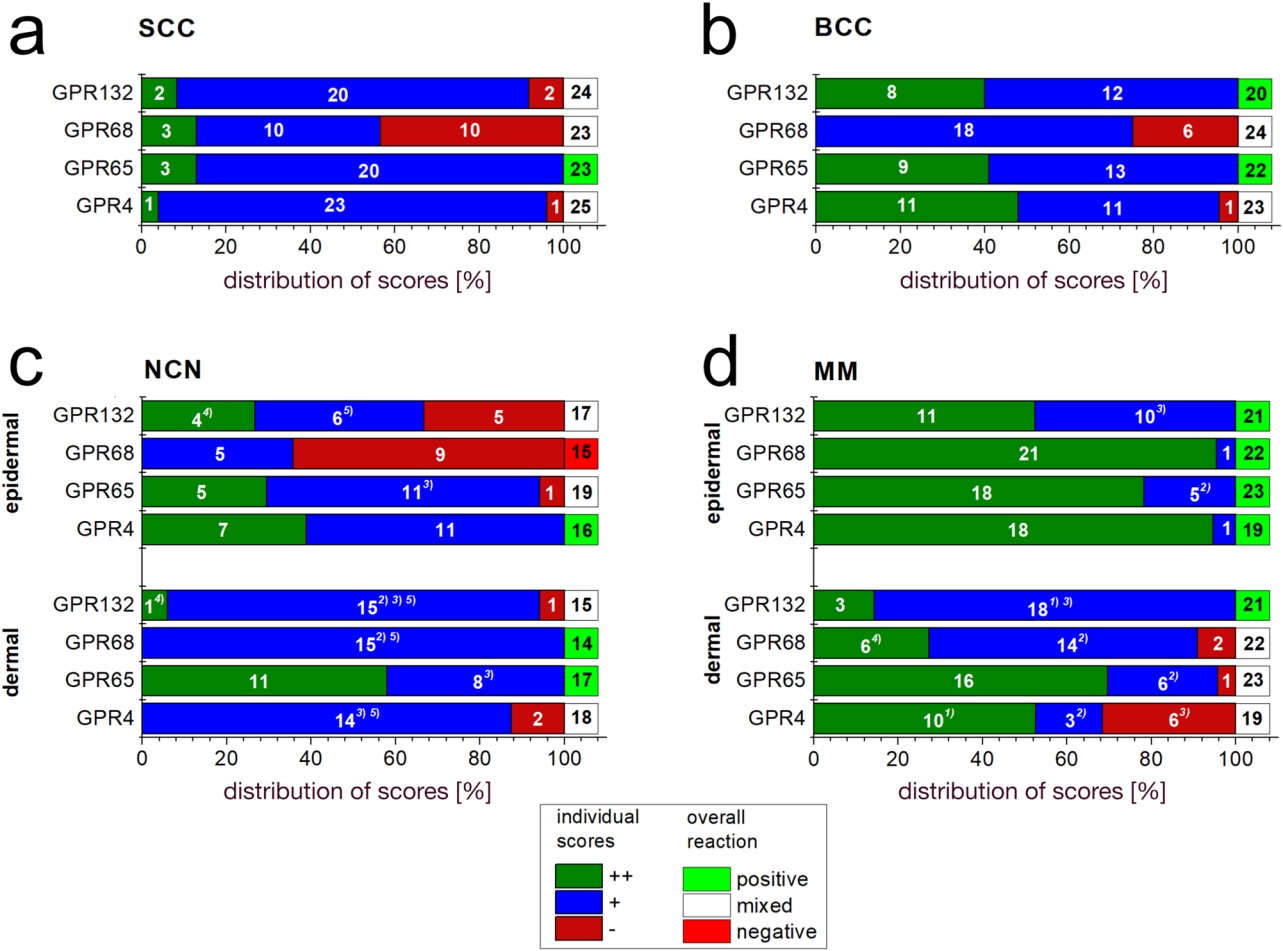
TMA-Score results for different pH-GPCRs in common skin tumors. Fractional distribution of the scores (green bar, ++: strong positive/positive reaction; +, blue bar: weak positive/ partial positive reaction; −, red bar: negative reaction) for GPR4 (GPR19), TDAG (GPR65), OGR1 (GPR68) and G2A (GPR132) of TMAs on different skin tumors (**a**) SCC, (**b**) BCC, (**c**) NCN, (**d**) MM. MM and NCN are further subclassified in epidermal and dermal portions. Numbers in bars indicate the occurrence of a respective score. The sum of all scores is 100%. Superscript numbers give information on scoring details: (1) strong positive either on the surface or in the deeper parts of the tumor tissue, (2) single tumor cells are strong positive, but the overall impression is weak positive, (3) large tumor cells appear strong positive, (4) partially strong positive and (5) weak positive. Overall evaluation is indicated by a green, white or red box (beyond 100% scale): green box: overall positive reactions; red box: overall negative reactions; white box: mixed reactions without clear majority in favour of one specific reaction. Numbers in this box indicate the total number of samples investigated. For additional information on the individual TMA score, see Supplementary Tables S2–S5.

### GPR4 (GPR19)

#### IHC and IF

According to the IHC data, 40% of the SCC tissue samples showed a strong GPR4 expression, while 40% of the samples were weak positive and 20% negative for GPR4 (Figs. 1a,e,i, 5a and Supplementary Figs. S1–S4 first column). For MM and NCN tumor tissue, epidermal and dermal tumor cells were distinguished in terms of GPR4 expression. Regarding the MM epidermal tissue, 20% of the samples strongly expressed GPR4, 40% were weak or partial positive and the remaining 40% showed no expression of GPR4. In contrast, the MM dermal sections were strong positive for GPR4 in 60% of the samples and weak or partial positive in 40% of the samples (Figs. 2a,e,i, 5d and Supplementary Figs. S5–S8 first column). 40% of NCN epidermal tissue showed a strong positive expression of GPR4, while the remaining 60% of the samples were only weak or partial positive. The dermal parts of the NCN tissue samples showed similar distribution of expression levels (Figs. 3a,e,i, 5c and Supplementary Figs. S9–S12 first column). BCCs strongly expressed GPR4 in only 16.6% of the cases, while the other 83.3% of BCC showed no expression (Figs. 4a,e,i, 5b and Supplementary Figs. S3–S17 first column).

#### The tissue microarray analysis (TMA)

TMA revealed that while only 4% of the SCC samples were strong positive for GPR4, 92% showed a weak GPR4 expression and 4% were GPR4 negative (Fig. 6a, Fig. S18). MM revealed strong positive results, especially in the epidermal areas of the tissue (94.7%) compared to the dermal section (52.6%) (Fig. 6d). NCN epidermal and dermal parts appeared both weak positive (epidermal: 61.1%, dermal: 87.5%) (Fig. 6c). In the dermal portions of MM and NCN especially the giant tumor cells appeared to be strong positive. 47.8% of BCC tissue samples strongly expressed GPR4 (Fig. 6b). Other TMA BCC tissue exhibited a weak positive expression (47.8%) or were negative for GPR4 (4.4%). For most instances, the TMA supported the trend of results described for the combined IHC and IF data, except of the BCC where one case not expressing GPR4 was observed in the TMA.

### TDAG8 (GPR65)

#### IHC and IF

Regarding the expression-profile of TDAG8 (GPR65), 20% of the SCC cells showed a strong positive expression, 80% seemed to express TDAG8 weaker or only partial (Figs. 1b,f,j, 5a and Supplementary Figs. S1–S4 second column). 60% of the MM epidermal sections showed a strong and 40% a weak TDAG8 expression, whereas 60% of the MM dermal sections were weak positive and 40% showed no expression at all (Figs. 2b,f,j, 5d and Supplementary Figs. S5–S8 second column). 40% of the NCN epidermal sections showed a strong positive expression of TDAG8. The other 60% exhibited a weak positive expression. TDAG8 occurrence in the NCN dermal part was similar to that in NCN epidermal portion (Figs. 3b,f,j, 5c and Supplementary Figs. S9–S12 second column). The BCC (Figs. 4b,f,j, 5b and Supplementary Figs. S13–S17 second column) showed a weak positive staining of TDAG8 in 33.3% of the cases and a negative staining in 66.7% of the samples.

#### TMA

In the TMA, 87% of the SCC samples showed a weak positive expression, supporting the other immunostaining results (Fig. 6a, Fig. S18). The majority of MM strongly expressed TDAG8, where the epidermal section was strong positive in 78.3% and the dermal part in 69.6% (Fig. 6d). In contrast, NCN tumors appeared strong positive (57.9%) in their dermal section, while the epidermal portion was predominantly only weak positive (64.7%) (Fig. 6c). Large NCN tumor cells were strong positive for TDAG8 in both, dermal and epidermal tissue. 40.9% of the BCC cells were strong positive and 59.1% were either partial or weak positive (Fig. 6b).

Overall, the TMA corresponded well with the previous results, although the TMA did not show any negative results for TDAG8 in BCC.

### OGR1 (GPR68)

#### IHC and IF

The evaluation of the OGR1 (GPR68) expression-profiles based on IHC showed that 20% of SCC tissue samples were weak positive for OGR1, while the other 80% showed no expression of OGR1 (Figs. 1c,g,k, 5a, and Supplementary Figs. S1–S4 third column). 40% of MM epidermal tissue samples strongly expressed OGR1, 40% were weak positive and the other 20% were negative for OGR1. In contrast, 80% of the MM dermal sections showed a partial or weak positive expression and 20% showed no expression (Fig. 2c,g,k, 5d and Supplementary Figs. S5–S8 third column). 60% of the NCN epidermal tissue samples were weak positive and the other 40% strongly expressed OGR1. Dermal areas in NCN tissue samples revealed strong expression of OGR1 in 20% of the samples, partial expression in 60% of the cases and no expression in 20% of the tested samples (Figs. 3c,g,k, 5c and Supplementary Figs. S9–S12 third column). 33.3% of the BCC samples (Figs. 4c,g,k, 5b and Supplementary Figs. S13–S17 third column) were weak positive and the other 66.6% were negative for OGR1.

#### TMA

13% of the SCC cells were strong positive and 43.5% were weak positive, while 43.5% did not express OGR1 at all (Fig. 6a, Fig. S18). The epidermal part of MM was mostly positive for OGR1 (95.5%), whereas the MM dermal section appeared predominantly weak positive (63.6%) (Fig. 6d). Dermal MM cells appear to be partially strong positive. 64.3% of the NCN epidermal portion was OGR1 negative, while the NCN dermal part reached 100% weak positive results (Fig. 6c). About 75% of the BCC cells expressed OGR1 positive, while 25% did not express this GPCR (Fig. 6b).

Taken together, the TMA correlated with the results of the IHC/IF except of the BCC. According to the TMA data, BCC were more likely positive for OGR1.

### G2A (GPR132)

#### IHC and IF

40% of the SCC tissue samples presented a clearly positive expression of G2A (GPR132), 40% appeared to be weak or partial positive and the other 20% showed no expression of G2A (Figs. 1d,h,l, 5a and Supplementary Figs. S1–S4 fourth column). In 20% of the samples, the MM epidermal sections showed a strong, in 60% a weak positive expression of G2A and in 20% of the cases there was no expression of G2A. In dermal areas of MM 20% of the MM samples strongly expressed G2A, while 80% showed a weak positive expression (Figs. 2d,h,l, 5d and Supplementary Figs. S5–S8 fourth column). Both NCN epidermal and dermal parts provided either strong or partial weak positive results: 40% of NCN samples were strong positive and 60% were weak positive for cells within the epidermal portion of NCN. In the case of the NCN dermal section the results were inverse: 60% showed strong positive expression of G2A, but only 40% were weak positive (Figs. 3d,h,l, 5c and Supplementary Figs. S9–S12 fourth column). Regarding the expression of G2A on BCC cells (Figs. 4d,h,l, 5b and Supplementary Figs. S13–S17 fourth column), about 50% of the tissue samples strongly expressed G2A and 50% of the specimen were partial positive.

#### TMA

83.4% of the SCC cells showed a weak G2A expression, 8.3% of the samples revealed strong positive expression and the other 8.3% were negative for G2A (Fig. 6a, Fig. S18). 52.4% of the MM epidermal section expressed G2A strongly and 85.7% of the dermal portion of MM had a weak positive expression (Fig. 6d). Giant MM tumor cells appeared to be strong positive in the epidermal and dermal parts. The NCN dermal part appeared to be mostly weak positive (88.2%) in contrast to the NCN epidermal zone, which revealed more negative results (33.3%) (Fig. 6c). 60% of the BCC cells expressed G2A weakly, whereas 40% of BCC showed strong expression (Fig. 6b). The TMA results fully confirmed the IHC/IF results.

## Discussion

In this study, we have examined the expression profiles of the pH-GPCRs GPR4 (GPR19), TDAG8 (GPR65), OGR1 (GPR68) and G2A (GPR132) on different types of common skin tumors, SCC, MM, NCN and BCC. Each tumor expresses typical sets of GPCRs: (1) GPR4 is expressed on all epidermal portions of NCN (IHC: 40% ++, 60% +, TMA: 38.9% ++, 61,1% +) and on epidermal (IHC: 20% ++, 40% +, TMA: 94.7% ++, 5.3% +) and dermal (IHC: 60% ++, 40% +, TMA: 52.6% ++, 15.8% +) MM. (2) G2A is clearly expressed on BCC (IHC: 50% ++, 50% +, TMA: 40% ++, 60% +) and the dermal MM area (IHC: 20% ++, 80% +, TMA: 14.3% ++, 85,7% +), while expression is also seen for epidermal MM portions (IHC: 20% ++, 40% +, TMA: 52.4% ++, 47.6% +) and NCN (mostly dermal). (3) TDAG8 is expressed on SCC (IHC: 20% ++, 80% +, TMA: 13% ++, 87% +), strongly on the epidermal MM (IHC: 60% ++, 40% +, TMA: 78,3% ++, 12.7% +) as well as the dermal portions of NCN (IHC: 40% ++, 60% +, TMA: 57,9% ++, 42.1% +) and (4) OGR1 is poorly expressed on SCC (IHC: 80%−, TMA: 43,5%−). It is striking, that all four GPCRs are particularly often expressed in NCN and MM. Mixed results were found for any other combination of pH-GPCR and tumor type (Figs. 5 and 6).

The very inhomogeneous expression-profiles support the current knowledge about the opposing roles of the four pH-GPCRs within a tumor^14^. The pH-GPCRs function as proton signal sensors and transducers^12^ and consequently, have an effect on cancer cell proliferation, metastasis, angiogenesis, apoptosis, immune cell function and inflammation, either in a pro-tumorigenic or in an anti-tumorigenic manner^12,23–25,27–29^. The role of the individual pH-GPCRs in tumor progression/regression and the impact of either overexpression or depletion of individual pH-GPCR types on different cell types is yet to be investigated.

### GPR4 (GPR19)

GPR4 is expressed on all epidermal NCN portions. Dermal NCN, MM, SCC and BCC varied in expression levels. While dermal NCN portions, SCC and BCC exhibited weak positive results, dermal MM showed strong positive results. Interestingly, the TMA stated a strong positive expression of GPR4 in the epidermal MM, and IHC/IF predominantly showed (strong) positive results for the epidermal portion of MM except of two tissue samples. GPR4 was found to be overexpressed in several human cancers^31^. GPR4-deficient mice showed a significantly reduced angiogenic response to VEGF, which accordingly led to a reduction in tumor growth in orthotopic models^29^. Acidification-activated GPR4 in endothelial cells increased the expression of a number of inflammatory genes and promoted angiogenesis in head and neck cancer, likely via secretion of angiogenic factors^32^. Regarding cancer cells themselves, it was shown that ectopic expression of GPR4 in murine 3T3 cells induced malignant transformation^33^. In contrast, GPR4 overexpression in B16F10 melanoma cells inhibited their acidic pH-induced migration, invasion and metastasis formation^22^. Taking this knowledge and our results into account, GPR4 might be an indicator of dysplasia of dermal melanocytes similar to HMB45. The latter is also found to often remain positive in deep dermal portions of dysplastic nevi or melanoma while expression levels decrease with increasing depth in normal nevi.

### TDAG8 (GPR65)

In all four tumor types investigated in this study expression of TDAG8 was high, except for dermal portions of MM, epidermal portions of NCN and BCC, where TDAG8 occurrence was often only moderate or missing. TDAG8 is predominantly expressed in lymphoid cells and tissues, including peripheral blood leukocytes, spleen, lymph nodes, and thymus and has also been detected in some selected cancers^31,34^. Overexpression of TDAG8 in lung carcinoma cells was associated with enhanced tumor development and cancer cell survival under acidic conditions^33^. Ectopic TDAG8 expression malignantly transformed a normal mammary epithelial cell line and led to ligand-independent activation of SRE and CRE promoter-driven gene transcription in HEK293 cells^31^. On the other hand, ectopic overexpression of TDAG8-GFP fusion protein enhanced apoptosis and sensitivity to dexamethasone-induced apoptosis in lymphoma cells^35^. TDAG8-deficiency in different KO mouse models was associated with an exacerbation of inflammation in selected pathologies^36–38^. In summary, it seems that TDAG8 attenuates immune-mediated inflammation, while the overall effect on non-blood-cell tumor cell behaviour remains less clear. Based on this knowledge, we hypothesize that the high expression of TDAG8 in the investigated skin tumors might be crucial for tumor growth and/or tumor cell survival. However, another possible mechanism could be that TDAG8 acts as a tumor suppressive receptor to control tumor growth under acidic conditions. These questions have to be addressed with cell culture experiments.

### OGR1 (GPR68)

Regarding the tumor tissue analysed in this study, OGR1 is not (IHC) or only moderately (TMA) expressed in SCC and BCC, but particularly present in MM and NCN. Overexpression of OGR1 in human prostate and ovarian cancer cells mediated an inhibitory effect on cell migration and metastasis^27,28,39^. In addition, OGR1 overexpression in ovarian cancer cells also inhibited cell proliferation, while increasing cell–matrix adhesion^27^, suggesting a tumor-supressing effect of ORG1. In contrast, when the host cells of ORG1 knock-out mice were depleted of ORG1 the tumorigenesis of injected melanoma cells and prostate cancer cells was decreased^40,41^, indicating tumor-promoting function of ORG1 in the host organism. In other cell types ORG1 expression and acid stimulation was associated with the expression of inflammatory and immune modulatory factors^42–45^. These findings state that the high expression of ORG1 in the mesenchymal tumors NCN and MM clearly differs from the expression in the epithelial tumors SCC and BCC. Further cell culture experiments are needed in order to study the exact effect of OGR1 in the skin.

### G2A (GPR132)

In skin G2A (GPR132) is proposedly expressed in keratinocytes, fibroblasts, epidermal cells and melanocytes^14^. The incidence of G2A in the investigated tumors was high in both, epidermal and dermal parts of MM, NCN and BCC, but less frequent in SCC. G2A is predominantly expressed by different immune cells^46,47^. G2A was identified as a stress-inducible gene, activated by genetic recombination processes in immature B lymphocytes and developing thymocytes, or by exposure to DNA-damaging stress, like UV, X-ray, etoposide or doxorubicin^48^. G2A expression led to cell cycle arrest and attenuated transformation potential of oncogenes^48^. The important role of G2A in controlling immune cell homeostasis was supported by the finding that G2A-deficiency caused autoimmune syndrome in ageing G2A-depleted mice^49^. However, expression of G2A in other cell types appears to have oncogenic potential, as high-level expression of G2A in NIH3T3 cells induced malignant transformation^50^. In human epidermal keratinocytes, G2A mediated the secretion of cytokines, and induced cell cycle arrest^46^. UVB radiation and H_2_O_2_ enhanced G2A expression in HaCaT cells, indicating that G2A might function as sensor for DNA damage and oxidative stress in keratinocytes^51^. With its high expression in skin tumors MM, NCN, SCC and BCC, G2A might play a pivotal role as an immune checkpoint of the tumor.

Regarding the expression of pH-GPCRs in different skin tumors, TMA results (Fig. 6) reveal that the overall expression of all four GPCRs increases in MM compared to NCN. Especially the incidence of strong positive expression of the pH-GPCRs is increased in both, dermal and, even more pronounced, in epidermal portions. Thus results suggest that an increase in pH-GPCR expression in MM could be a marker for increased malignancy, which requires, however, further investigation.

The prevailing hypothesis, that influencing factors such as type of cancer, the micro- and macro environment as well as the variation between every human individual influence individual pH-GPCR expression, can be supported with the results of this study, containing a large data set for the four most common skin cancers.

In summary, the current evidence on the expression of pH-GPCRs in tumors is still only a first step towards understanding the role of pH-GPCRs and their function as transmembrane messengers of extracellular pH in cancer development or control. Further functional studies are undoubtedly required to fully understand the individual role of each pH-GPCR in the development and progression of different skin cancers.

The cancer type-specific differential expression of individual pH-GPCRs underpins their potential value in the field of cancer therapy. Our investigations may lead to more specific cell culture studies of the pH-GPCRs in different skin tumor cell lines and their use as a potential therapeutic target.

## Materials and methods

### Tissue samples

For all experiments, we used tissue samples older than 10 years from the department of Dermatology at the University Medical Center Regensburg (IHC/IF: n = 5, exception: BCC n = 6; TMA: n = 24–27). Routine paraffin-embedded skin biopsies obtained from affected areas of patients with localized skin tumors were used anonymized. The diagnosis of localized tumors had been previously confirmed histologically by a dermatopathologist. Handling of human skin tumor biopsies older than 10 years was approved by the ethical committee of the University of Regensburg. Under German law the tumor tissue left after surgery after the final diagnosis can be discarded after 10 years or are free to use.

General data regarding the tissue sample origin is given in Supplementary Table S1. The following tissue types served as positive controls: primary human tonsil tissue and lung tissue for G2A and TDAG8, endosomal membrane of the testis and lung for OGR1 and pancreas as well as the endosomal membrane of the lung for GPR4. As negative control tissue we used liver for GPR4, heart muscle for GPR65, pancreas for GPR68 and ovary for GPR132. Respective images from IHC staining on control tissue including secondary antibody controls and isotype antibody controls are shown in supplementary figure S19. Additional antibody controls on dermal tumor tissue are shown in supplementary figure S20.

### Immunohistochemistry (IHC)

Hematoxylin and eosin (HE)-stained, paraffin-embedded and fixed tissues as well as positive and negative controls were freshly cut into 2 μm thin pieces and superimposed on slides. Tissue sections were incubated for 30 min at 72 °C before they were rehydrated by washing with alcohol solutions at descending concentrations as follows: 2 × xylol for 5 min, 2 × 100% ethanol for 5 min, 2 × 96% Ethanol for 5 min, 2 × 70% ethanol for 5 min. Endogenous peroxidase was neutralised by incubation with 3% H_2_O_2_ (Fisher Scientific GmbH, Schwerte, Germany) for 10 min. Afterwards, the slides were washed in distilled water and were submersed in precooked citrate buffer (boiled for 30 min) (Zytomed Systems GmbH, Berlin, Germany) for 20 min. After cooling the sections on ice, they were incubated in phosphate-buffered saline (PBS) (Sigma-Aldrich, St. Louis, United States of America) for 10 min at RT. Subsequently, sections were clamped to Shandon coverplate immunostaining chambers Fisher Scientific GmbH, Schwerte, Germany) and transferred to PBS. Samples were incubated for 10 min at RT in a blocking solution (ZytoChem Plus HRP Kit/Rabbit, Zytomed Systems GmbH, Berlin, Germany) in order to minimise unspecific binding of antibodies.

In the following, tissue sections were treated with polyclonal primary antibody (rabbit anti-human GPR4 (1:200; Abcam, Cambridge, Great Britain, Anti-GPCR GPR4 antibody, ab188606), GPR65 (1:500; Abcam, Cambridge, Great Britain, Anti-GPCR GPR65 antibody, ab188907), GPR68 (1:50; Abcam, Cambridge, Great Britain, Anti-OGR1 antibody, ab188964) and GPR 132 (1:60; Abcam, Cambridge, Great Britain, Anti-GPCR G2A antibody, ab116586) or isotype control antibody (1:200, Abcam, Cambridge, Great Britain, rabbit IgG polyclonal isotype control, ab27478) in antibody diluent (Zytomed Systems GmbH, Berlin, Germany) overnight at 4 °C. The following day, sections were rinsed in PBS (3 × 5 min) and then incubated with the secondary biotinylated antibody (ZytoChem Plus HRP Kit/Rabbit, Zytomed Systems GmbH, Berlin, Germany) for 30 min at RT. After three washes with PBS, samples were incubated with streptavidin-HRP-conjugate for 20 min at RT, followed by another washing step with PBS. Finally, chromogen solution AEC plus (Dako, Glostrup, Denmark), was added. The reaction was stopped by several washes with distilled water as soon as the positive controls showed distinct staining. Mayer’s Haemalm (Carl Roth GmbH & Co., Karlsruhe, Germany) was used to counterstain the tissue. Samples were embedded with Aquetex mounting medium (Merck KGaA, Darmstadt, Germany).

Specimen were inspected with a Leitz Wild Biomed microscope (Leica Microsystems GmbH, Wetzlar, Germany, Type: 020-507.010) and afterwards scanned with the PreciPoint M8. Digital images were edited using the analysis software ViewPoint online (PreciPoint, Freising, Germany). Images were evaluated via visual inspection. Scores were assigned for ++: strong positive/positive reaction; +: weak positive/partial positive reaction; −: negative reaction.

### Tissue microarray (TMA)

The immunohistochemical multiple-labelling tissue microarray (TMA) allows for simultaneous IHC staining of multiple tissue samples. Representative tumor material from 24–27 tissue samples per tumor type was assembled into a paraffin matrix (5 × 6) with 1 mm diameter spots. Samples on the TMA tissue slide were subjected to IHC staining following the protocol above.

### Immunofluorescence (IF)

Samples were incubated in the heating cabinet for 20 min at 70 °C and rehydrated with the descending order of the alcohol concentration as described above. The slides were washed with PBS and subsequently incubated in citrate-tris-EDTA-buffer (Zytomed Systems GmbH, Berlin, Germany) for 25 min. The sections were cooled on ice for 25 min and then submersed in PBS for 10 min. The samples were incubated for 15 min in tris-glycine-buffer (Trishydroxymethylaminomethan: Merck KGaA, Darmstadt, Germany; Glycine: Merck KGaA, Darmstadt, Germany) to reduce autofluorescence. After three rinses with PBS, samples were incubated with 5% BSA (Sigma-Aldrich, St. Louis, United States of America) in tris-buffered saline with 0.1% Tween-20 (TBST) (Tween-20: Carl Roth GmbH & Co., Karlsruhe, Germany) for 60 min in order to block unspecific binding of antibodies. Tissue sections were incubated with primary antibody (GPR4 1:100, GPR65 1:300; GPR68 1:250, GPR132 1:60) in phosphate-buffered saline 1% Tween-20 (PBST) at 4 °C overnight. Afterwards, samples were washed three times for 15 min. Alexa-594-conjugated goat anti-rabbit specific second antibody (Life Technologies, Carlsbad, United States of America, A11037) was diluted with 1% BSA in PBST (1:1,000) and added to the slides for 30 min. Afterwards, tissue sections were rinsed once with PBS for 15 min, and finally stained for cell nuclei with 4,5-diamindino-2-phenylindole (DAPI). Fluorescence was exposed with a Zeiss Axio Imager.

### Scoring

Scoring was based on visual assessment of cell number and intensity of staining. The grades were: ++: strong positive/positive histochemical reaction, with > 80% of cells positive and/or high staining intensity; +: weak positive/ partial positive reaction, with 20—80% of cells positive and staining weak or only partially strong; −: negative reaction, with < 20% cells with weak staining. Assessment was done by two experienced histopathologists. Further comments were made if necessary: (1) strong positive either on the surface or in the deeper parts of the tumor tissue, (2) single tumor cells are strong positive, but the overall impression is weak positive, (3) large tumor cells appear strong positive, (4) partially strong positive and (5) weak positive.

### Ethics

All experiments were done in accordance with the declaration of Helsinki. No identifying data of tissue donors were used during experiments or in the paper. All tissue samples were from patients older than 18 years.

## Supplementary information

Supplementary information.
