# Supplementary material for: Expression profiles of proton-sensing G-protein coupled receptors in common skin tumors
Source: Sci Rep. 2020 Sep 18;10:15327. doi: 10.1038/s41598-020-71700-9 (PMC7501253; doi:10.1038/s41598-020-71700-9)
Supplement: Supplementary file 1 — Supplementary information. [file 41598_2020_71700_MOESM1_ESM.pdf]

Supplementary Information

Expression profiles of Proton-sensing G-Protein coupled receptors in common skin tumors

Wybke Klatt, Susanne Wallner, Christoph Brochhausen, Judith A. Stolwijk, Stephan Schreml*

Supplementary Figures

Figure S1

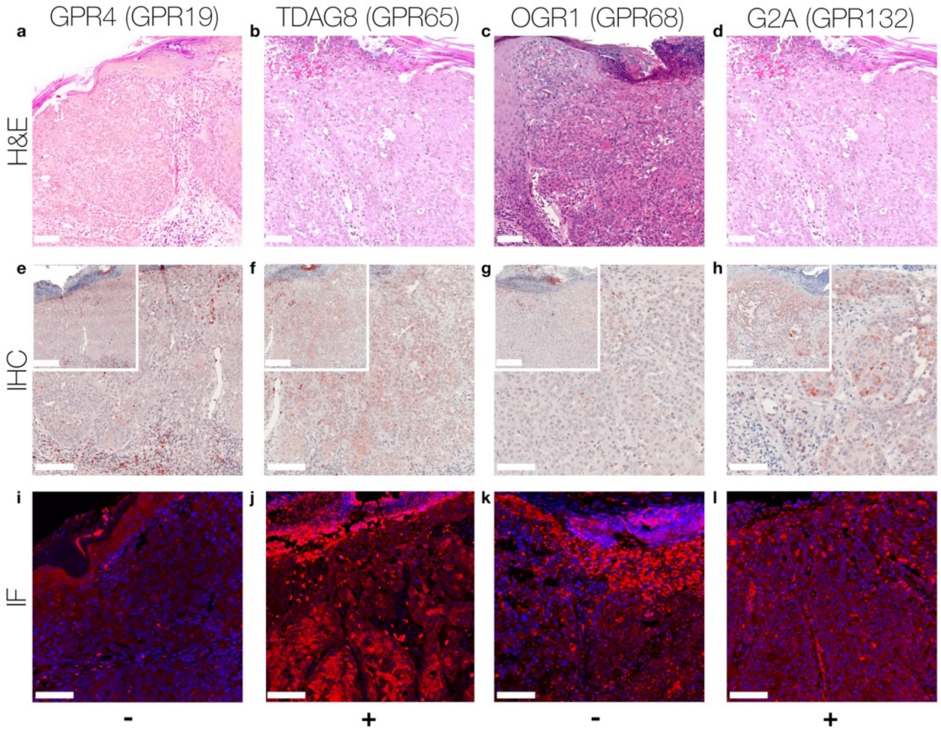

Figure S1: Immunohistochemistry and Immunofluorescence of SCC.
Immunohistochemical and immunofluorescent staining for GPR4 (GPR19), TDAG8 (GPR65), OGR1 (GPR68) and G2A (GPR132) in SCC tissue. (a-d) histochemical H&E staining, (e-h) immunohistochemical staining, inserted images present a 2x larger field of view, (i-l) immunofluorescence staining, red: secondary antibody label, blue: DAPI. Scale bars correspond to 100 μm (a-l: patient 2). Scores (bottom row) were assigned for ++: strong positive/positive reactions; +: weak positive/partial positive reaction; -: negative reaction. The SCC shows no expression of GPR4 and OGR1. There is a weak positive expression of TDAG8 and G2A.

Figure S2

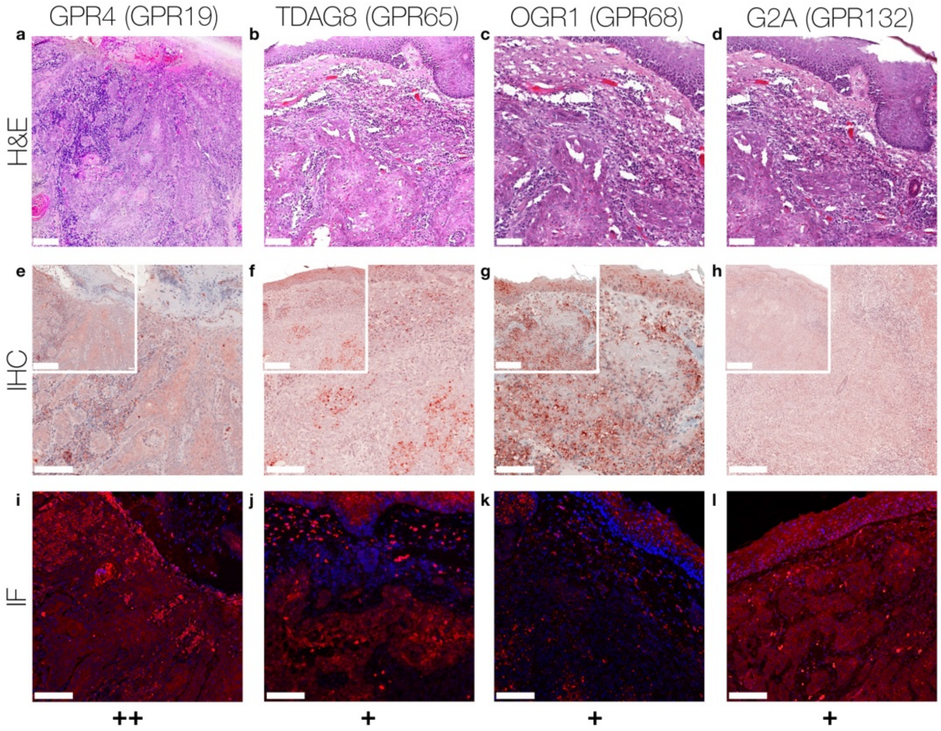

Figure S2: Immunohistochemistry and Immunofluorescence of SCC.
Immunohistochemical and immunofluorescent staining for GPR4 (GPR19), TDAG8 (GPR65), OGR1 (GPR68) and G2A (GPR132) in SCC tissue. (a-d) histochemical H&E staining, (e-h) immunohistochemical staining, inserted images present a 2x larger field of view, (i-l) immunofluorescence staining, red: secondary antibody label, blue: DAPI. Scale bars correspond to 100 μm (a, e, i: patient 1; b, c, d, f, g, h, j, k, l: patient 3). Scores (bottom row) were assigned for ++: strong positive/positive reactions; +: weak positive/partial positive reaction; -: negative reaction. The SCC shows a weak positive expression of TDAG8, OGR1 and G2A. GPR4 is expressed strongly on tumour cells.

Figure S3

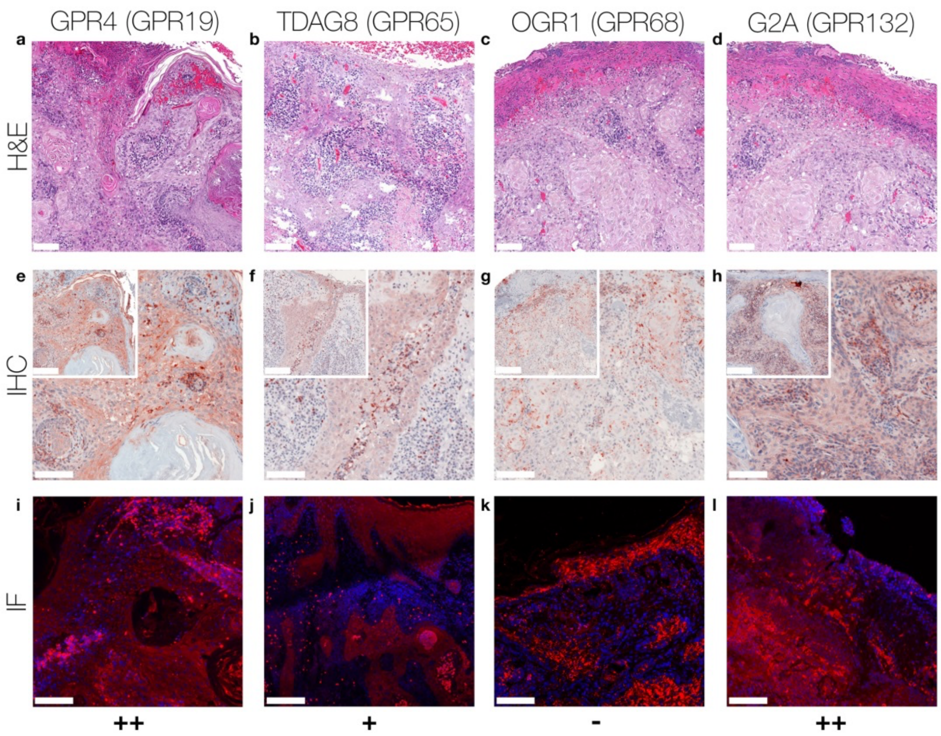

Figure S3: Immunohistochemistry and Immunofluorescence of SCC.
Immunohistochemical and immunofluorescent staining for GPR4 (GPR19), TDAG8 (GPR65), OGR1 (GPR68) and G2A (GPR132) in SCC tissue. (a-d) histochemical H&E staining, (e-h) immunohistochemical staining, inserted images present a 2x larger field of view, (i-l) immunofluorescence staining, red: secondary antibody label, blue: DAPI. Scale bars correspond to 100 μm (a, c, d, e, g, h, i, k, l: patient 4, b, f, j: patient 5). Scores (bottom row) were assigned for ++: strong positive/positive reactions; +: weak positive/partial positive reaction; -: negative reaction. The SCC shows no expression of OGR1. The tumor cells show a partial positive expression of TDAG8. The expression of GPR4 and G2A is significantly increased on tumor cells.

Figure S4

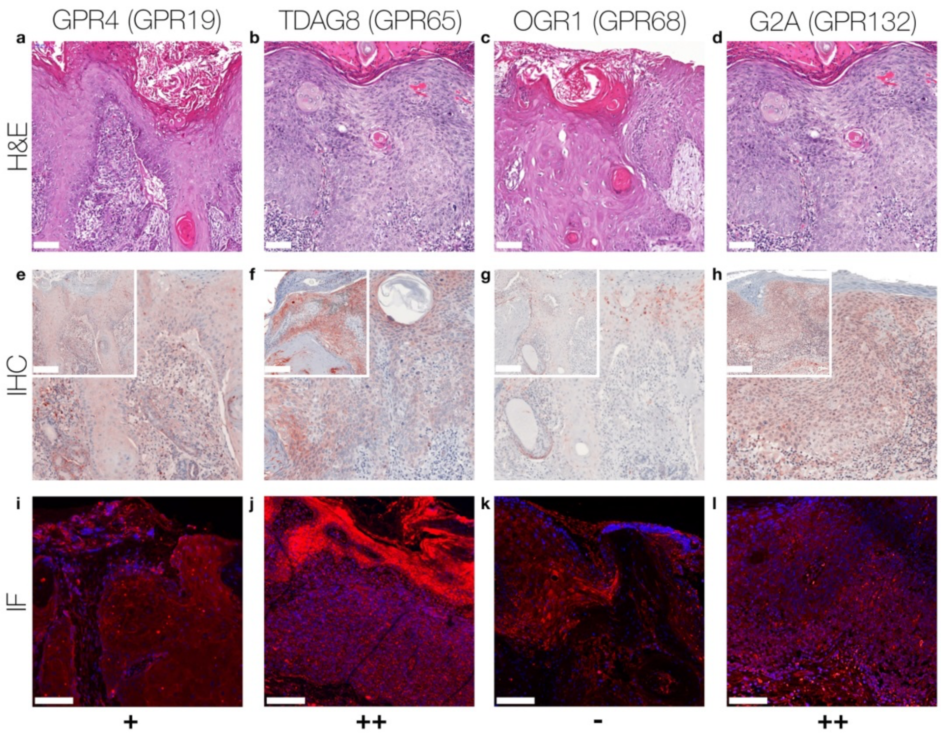

Figure S4: Immunohistochemistry and Immunofluorescence of SCC.
Immunohistochemical and immunofluorescent staining for GPR4 (GPR19), TDAG8 (GPR65), OGR1 (GPR68) and G2A (GPR132) in SCC tissue. (a-d) histochemical H&E staining, (e-h) immunohistochemical staining, inserted images present a 2x larger field of view, (i-l) immunofluorescence staining, red: secondary antibody label, blue: DAPI. Scale bars correspond to 100 μm (a, c, e, g, i: patient 6; b, d, f, h, j, l: patient 7). Scores (bottom row) were assigned for ++: strong positive/positive reactions; +: weak positive/partial positive reaction; -: negative reaction. The SCC shows no expression of OGR1 and a weak positive expression of GPR4. It shows a significantly increased expression of TDAG8 and G2A on the surface of tumor cells.

Figure S5

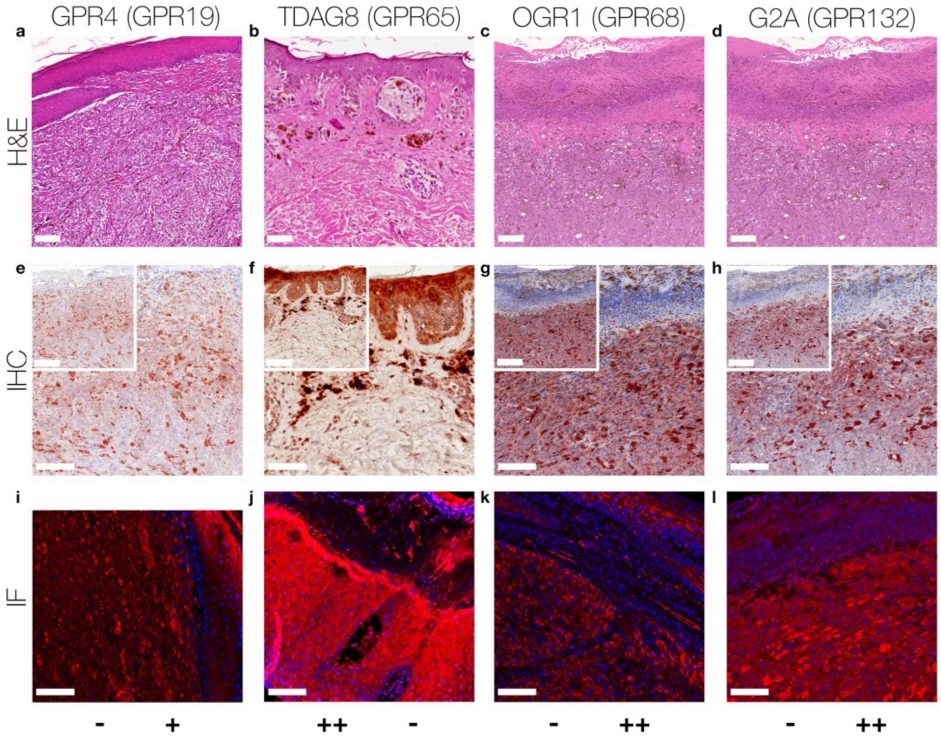

Figure S5: Immunohistochemistry and Immunofluorescence of MM.

Immunohistochemical and immunofluorescent staining for GPR4 (GPR19), TDAG8 (GPR65), OGR1 (GPR68) and G2A (GPR132) in MM tissue. (a-d) histochemical H&E staining, (e-h) immunohistochemical staining, inserted images present a 2x larger field of view, (i-l) immunofluorescence staining, red: secondary antibody label, blue: DAPI. Scale bars correspond to 100 μm (b, f, j: patient 10; a, c, d, e, g, h, k, i, l: patient 11). Scores (bottom row) were assigned for ++: strong positive/positive reactions; +: weak positive/partial positive reaction; -: negative reaction for the epidermal (left score) and the dermal (right score) region. The MM shows no expression on the epidermal regions of GPR4, OGR1 and G2A as well as there is a strong expression on the epidermal part of TDAG8. GPR4 is expressed weakly in the dermis, whereas TDAG8 is not expressed in this section. The dermis of OGR1 and G2A is strongly expressed. It has to be mentioned, that especially in those last two mentioned GPCRs smaller tumor cells appear to be weak positive and multinuclear giant cells are expressed more strong.

Figure S6

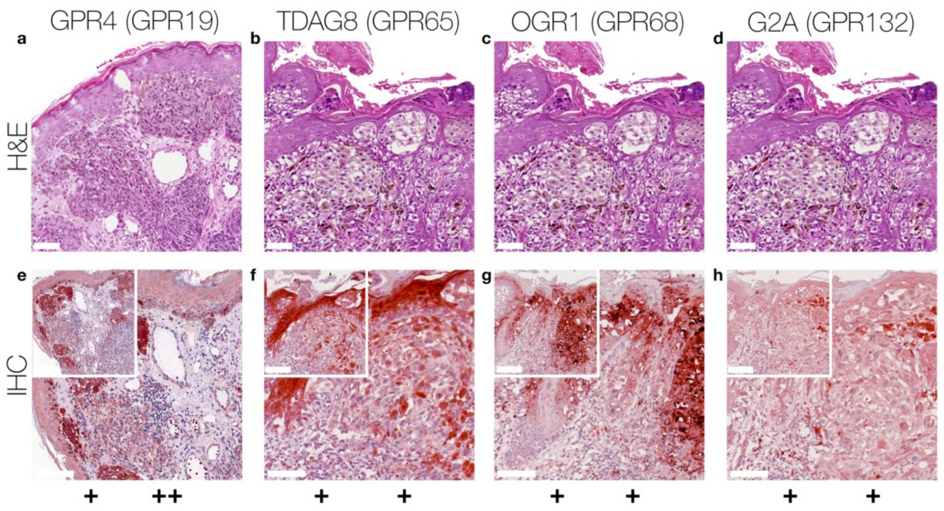

Figure S6: Immunohistochemistry and of MM.

Immunohistochemical and immunofluorescent staining for GPR4 (GPR19), TDAG8 (GPR65), OGR1 (GPR68) and G2A (GPR132) in MM tissue. (a-d) histochemical H&E staining, (e-h) immunohistochemical staining, inserted images present a 2x larger field of view. Scale bars correspond to 100 μm (a-l: patient 12). Scores (bottom row) were assigned for ++: strong positive/positive reactions; +: weak positive/partial positive reaction; -: negative reaction for the epidermal (left score) and the dermal (right score) region. The MM shows a partial positive expression on the epidermal and dermal regions of TDAG8, OGR1 and G2A. GPR4 is expressed weakly in the epidermis, but strongly in the dermis. Some tumor nest are only weakly coloured, others with multinuclear giant cells are expressed strongly.

Figure S7

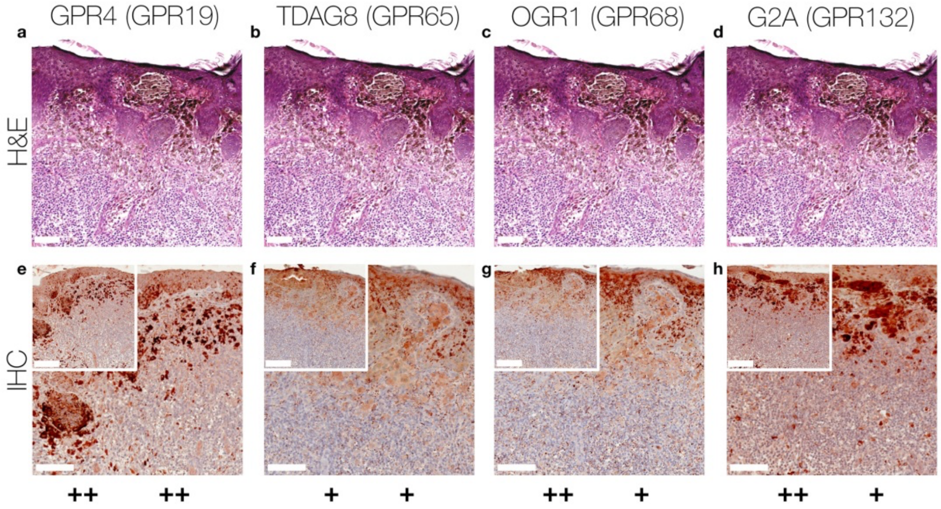

Figure S7: Immunohistochemistry of MM.

Immunohistochemical and immunofluorescent staining for GPR4 (GPR19), TDAG8 (GPR65), OGR1 (GPR68) and G2A (GPR132) in SCC tissue. (a-d) histochemical H&E staining, (e-h) immunohistochemical staining, inserted images present a 2x larger field of view (a-l: patient 13). Scores (bottom row) were assigned for ++: strong positive/positive reactions; +: weak positive/partial positive reaction; -: negative reaction for the epidermal (left score) and the dermal (right score) region. There is a strong positive epidermal expression of GPR4, OGR1 and G2A detected. TDAG8 is weak positive epidermally. The dermal expression of TDAG8, OGR1 and G2A is partial positive, but the GPR4 is expressed strongly in the dermal area. Smaller tumor cells within the tumor appear to be weak positive, whereas multinuclear giant tumor cells are strongly expressed.

Figure S8

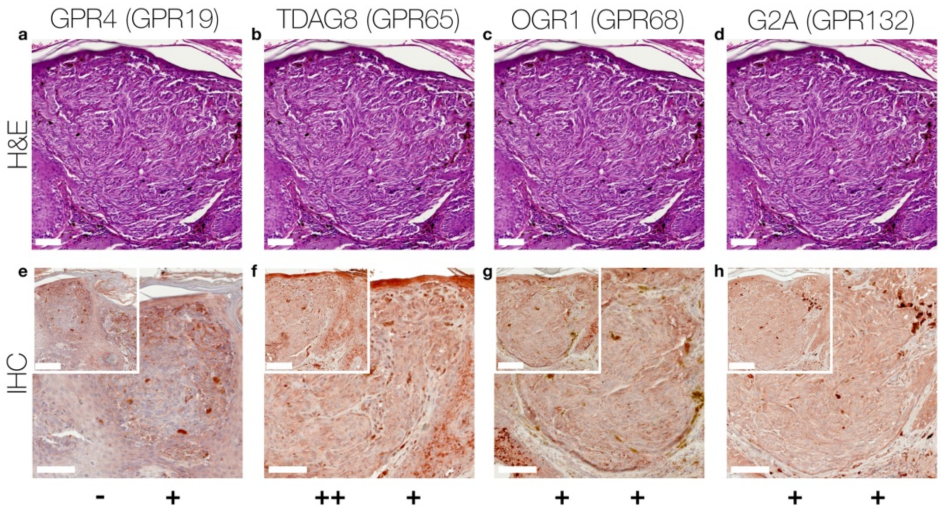

Figure S8: Immunohistochemistry of MM.

Immunohistochemical and immunofluorescent staining for GPR4 (GPR19), TDAG8 (GPR65), OGR1 (GPR68) and G2A (GPR132) in MM tissue. (a-d) histochemical H&E staining, (e-h) immunohistochemical staining, inserted images present a 2x larger field of view (a-l: patient 14). Scores (bottom row) were assigned for ++: strong positive/positive reactions; +: weak positive/partial positive reaction; -: negative reaction for the epidermal (left score) and the dermal (right score) region. The MM shows no epidermal expression of GPR4, but TDAG8 is strongly expressed in the epidermis. OGR1 and G2A are partial positive regarding the epidermis. All four GPCRs are expressed partially in the dermis. Smaller tumor cells within the tumor appear to be weak positive, while multinuclear giant tumor cells with altered nucleus-cytoplasmic ratio are strongly expressed.

Figure S9

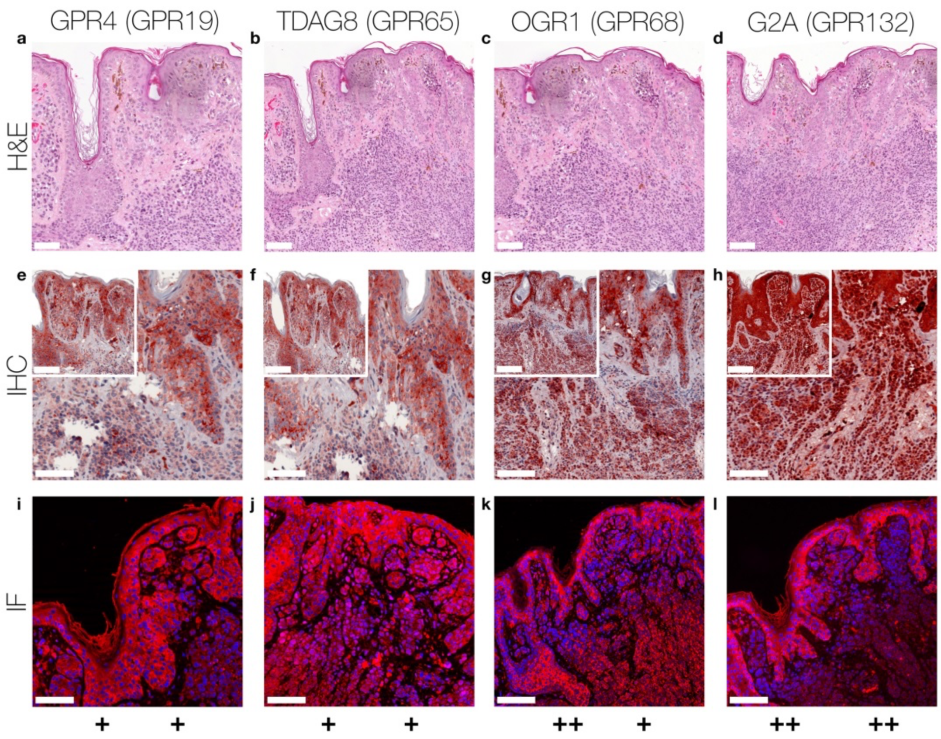

Figure S9: Immunohistochemistry and Immunofluorescence of NCN.
Immunohistochemical and immunofluorescent staining for GPR4 (GPR19), TDAG8 (GPR65), OGR1 (GPR68) and G2A (GPR132) in NCN tissue. (a-d) histochemical H&E staining, (e-h) immunohistochemical staining, inserted images present a 2x larger field of view, (i-l) immunofluorescence staining, red: secondary antibody label, blue: DAPI. Scale bars correspond to 100 μm (a-l: patient 16). Scores (bottom row) were assigned for ++: strong positive/positive reactions; +: weak positive/partial positive reaction; -: negative reaction for the epidermal (left score) and the dermal (right score) region. The NCN shows a strong positive expression on the epidermal regions of OGR1 and G2A. The epidermal expression of GPR4 and TDAG8 is partial positive. Besides the strong positive dermal expression of GPR132, the other three receptors GPR4, TDAG8 and OGR1 are weak positive in the dermis.

Figure S10

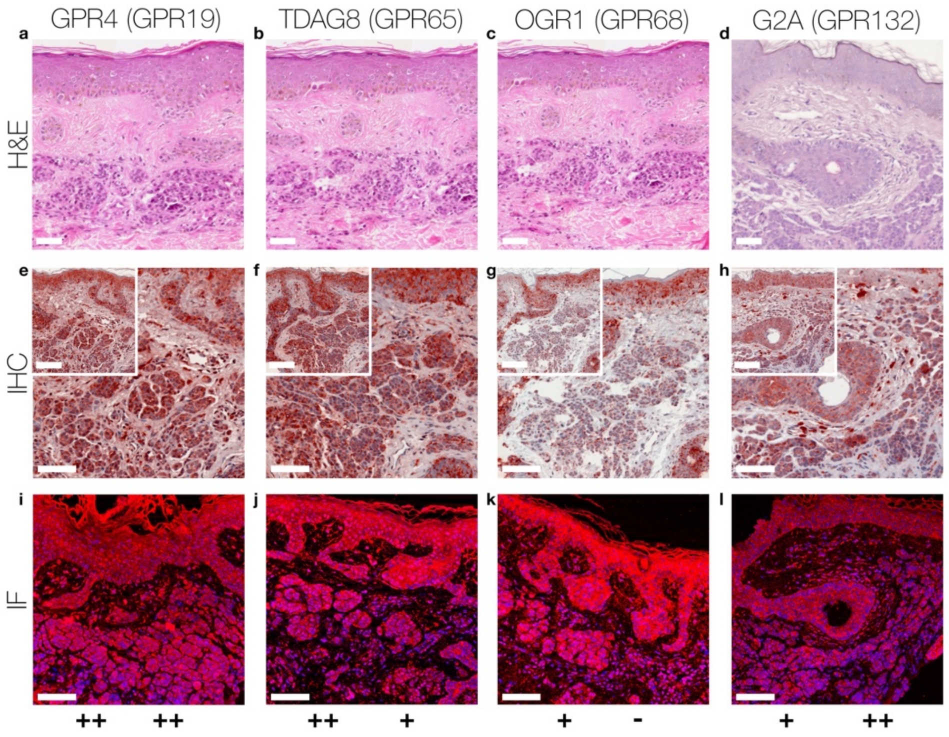

Figure S10: Immunohistochemistry and Immunofluorescence of NCN.
Immunohistochemical and immunofluorescent staining for GPR4 (GPR19), TDAG8 (GPR65), OGR1 (GPR68) and G2A (GPR132) in NCN tissue. (a-d) histochemical H&E staining, (e-h) immunohistochemical staining, inserted images present a 2x larger field of view, (i-l) immunofluorescence staining, red: secondary antibody label, blue: DAPI. Scale bars correspond to 100 μm (a-l: patient 17). Scores (bottom row) were assigned for ++: strong positive/positive reactions; +: weak positive/partial positive reaction; -: negative reaction for the epidermal (left score) and the dermal (right score) region. The epidermal expression of GPR4 and TDAG8 is significantly increased on NCN. There is a weak expression of OGR1 and G2A in the epidermis. In contrast, there is no dermal expression of OGR1 and only a weak expression of TDAG8. GPR4 and G2A are significantly expressed with an overall stronger expression on multinuclear giant cells in the dermis than smaller tumor cells within the tumor.

Figure S11

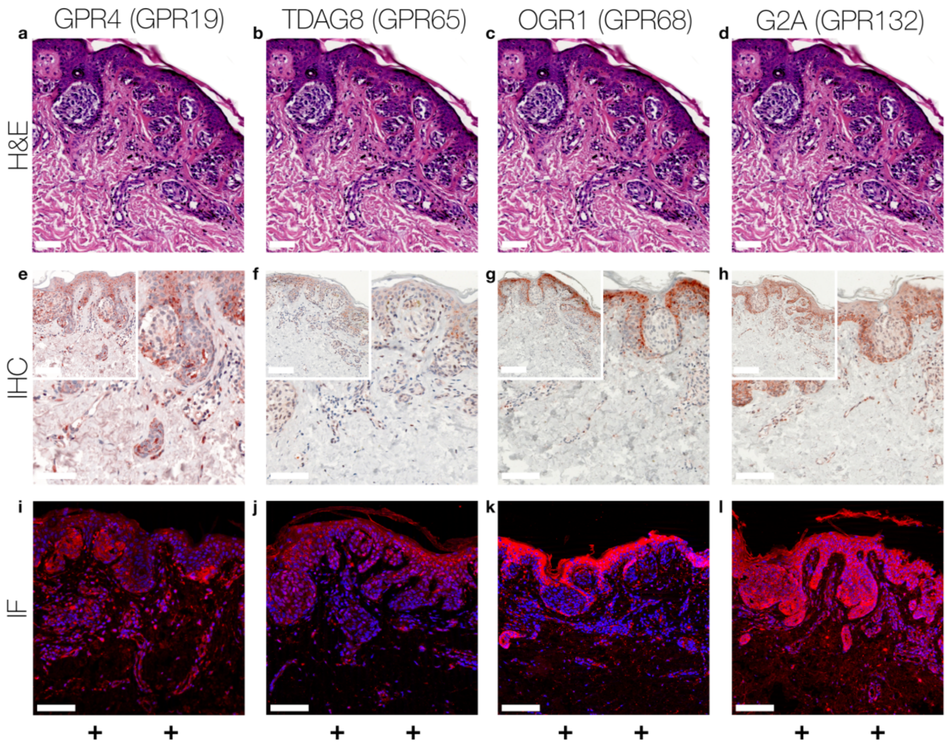

Figure S11: Immunohistochemistry and Immunofluorescence of NCN.

Immunohistochemical and immunofluorescent staining for GPR4 (GPR19), TDAG8 (GPR65), OGR1 (GPR68) and G2A (GPR132) in NCN tissue. (a-d) histochemical H&E staining, (e-h) immunohistochemical staining, inserted images present a 2x larger field of view, (i-l) immunofluorescence staining, red: secondary antibody label, blue: DAPI. Scale bars correspond to 100 μm (a-l: patient 18). Scores (bottom row) were assigned for ++: strong positive/positive reactions; +: weak positive/partial positive reaction; -: negative reaction for the epidermal (left score) and the dermal (right score) region. The NCN sample shows a weak positive epidermal as well as dermal expression of all four GPCRs. Especially in the dermal part of GPR4 smaller tumor cells within the tumor appear to be weak positive for GPR4 and multinuclear giant tumor cells with altered nucleus-cytoplasmic ratio strongly express GPR4.

Figure S12

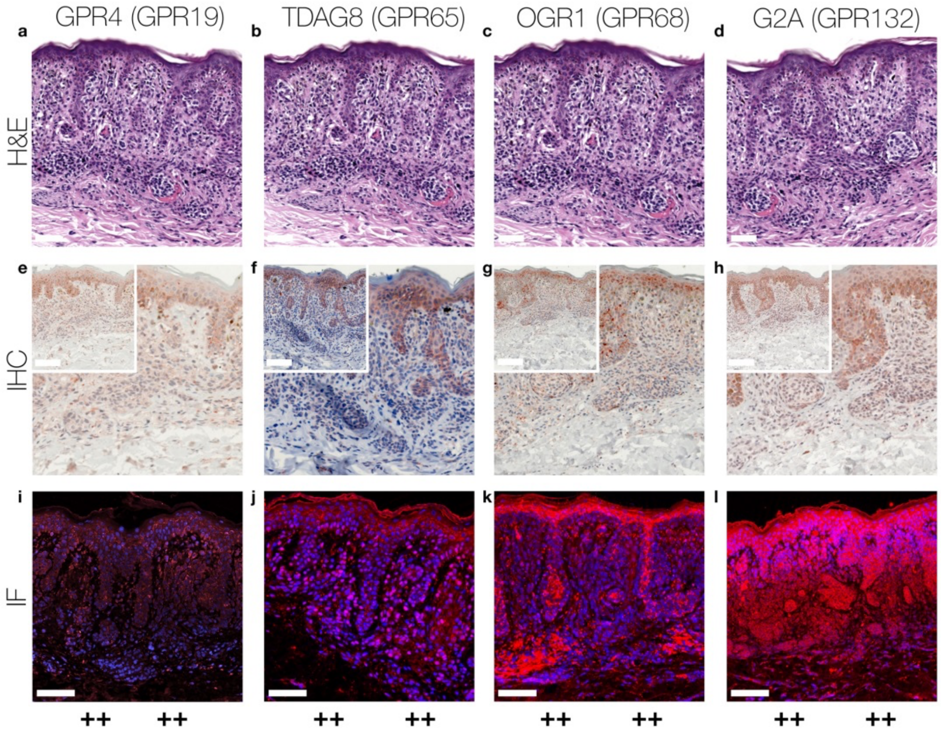

Figure S12: Immunohistochemistry and Immunofluorescence of NCN.
Immunohistochemical and immunofluorescent staining for GPR4 (GPR19), TDAG8 (GPR65), OGR1 (GPR68) and G2A (GPR132) in NCN tissue. (a-d) histochemical H&E staining, (e-h) immunohistochemical staining, inserted images present a 2x larger field of view, (i-l) immunofluorescence staining, red: secondary antibody label, blue: DAPI. Scale bars correspond to 100 μm (a-l: patient 19). Scores (bottom row) were assigned for ++: strong positive/positive reactions; +: weak positive/partial positive reaction; -: negative reaction for the epidermal (left score) and the dermal (right score) region. The NCN shows a strong positive epidermal as well as dermal expression of all four GPCRs.

Figure S13

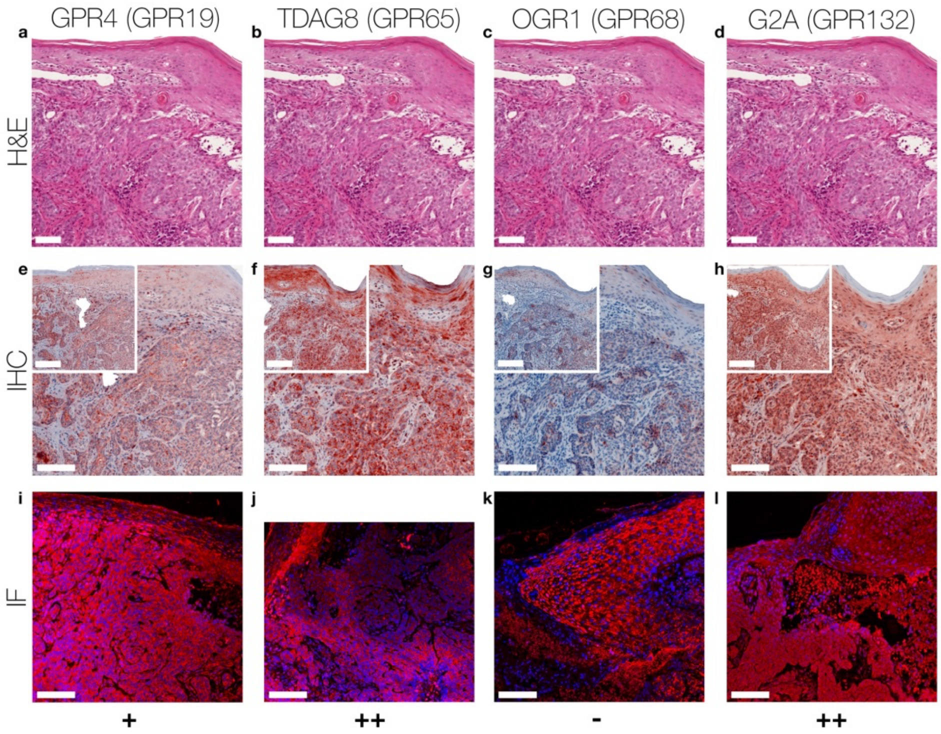

Figure S13: Immunohistochemistry and Immunofluorescence of BCC.
Immunohistochemical and immunofluorescent staining for GPR4 (GPR19), TDAG8 (GPR65), OGR1 (GPR68) and G2A (GPR132) in BCC tissue. (a-d) histochemical H&E staining, (e-h) immunohistochemical staining, inserted images present a 2x larger field of view, (i-l) immunofluorescence staining, red: secondary antibody label, blue: DAPI. Scale bars correspond to 100 μm (a-l: patient 21). Scores (bottom row) were assigned for ++: strong positive/positive reactions; +: weak positive/partial positive reaction; -: negative reaction. The expression of TDAG8 and G2A is significantly increased on BCC. The BCC shows a weak positive expression of GPR4.There is no expression of OGR1.

Figure S14

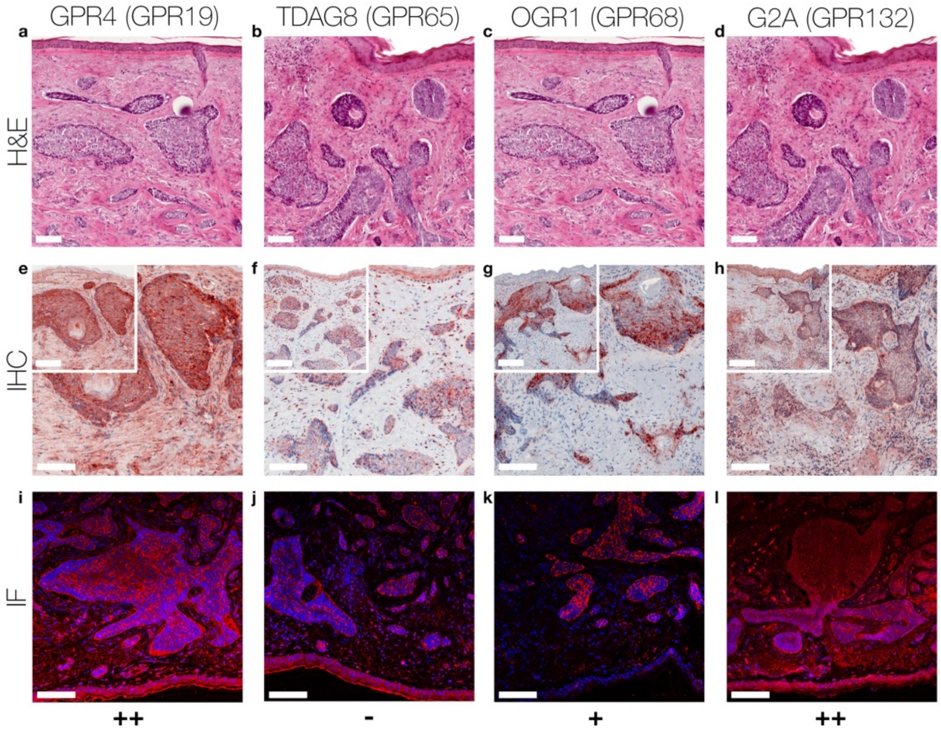

Figure S14: Immunohistochemistry and Immunofluorescence of BCC.
Immunohistochemical and immunofluorescent staining for GPR4 (GPR19), TDAG8 (GPR65), OGR1 (GPR68) and G2A (GPR132) in BCC tissue. (a-d) histochemical H&E staining, (e-h) immunohistochemical staining, inserted images present a 2x larger field of view, (i-l) immunofluorescence staining, red: secondary antibody label, blue: DAPI. Scale bars correspond to 100 μm (a-l: patient 22). Scores (bottom row) were assigned for ++: strong positive/positive reactions; +: weak positive/partial positive reaction; -: negative reaction. The BCC shows on the one hand a significantly increased expression of GPR4 as well as G2A and on the other hand a weak positive expression of OGR1. In contrast, there is no expression of TDAG8, only several peritumoral lymphocytes appear to be positive.

Figure S15

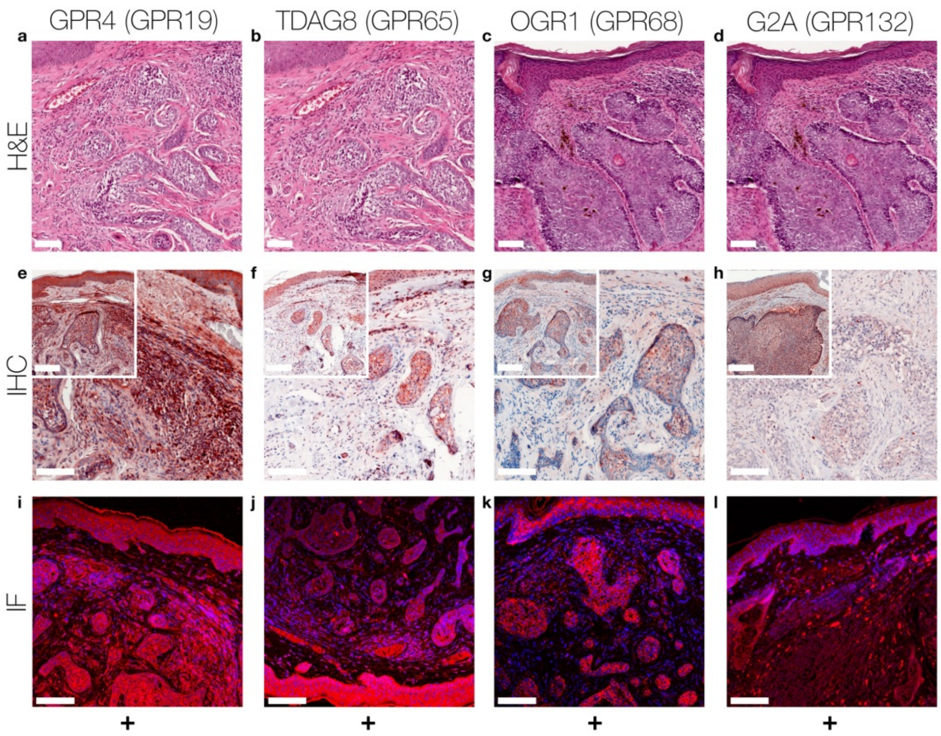

Figure S15: Immunohistochemistry and Immunofluorescence of BCC.
Immunohistochemical and immunofluorescent staining for GPR4 (GPR19), TDAG8 (GPR65), OGR1 (GPR68) and G2A (GPR132) in BCC tissue. (a-d) histochemical H&E staining, (e-h) immunohistochemical staining, inserted images present a 2x larger field of view, (i-l) immunofluorescence staining, red: secondary antibody label, blue: DAPI. Scale bars correspond to 100 μm (a-l: patient 23). Scores (bottom row) were assigned for ++: strong positive/positive reactions; +: weak positive/partial positive reaction; -: negative reaction. The BCC shows a weak positive expression of all four GPCRs.

Figure S16

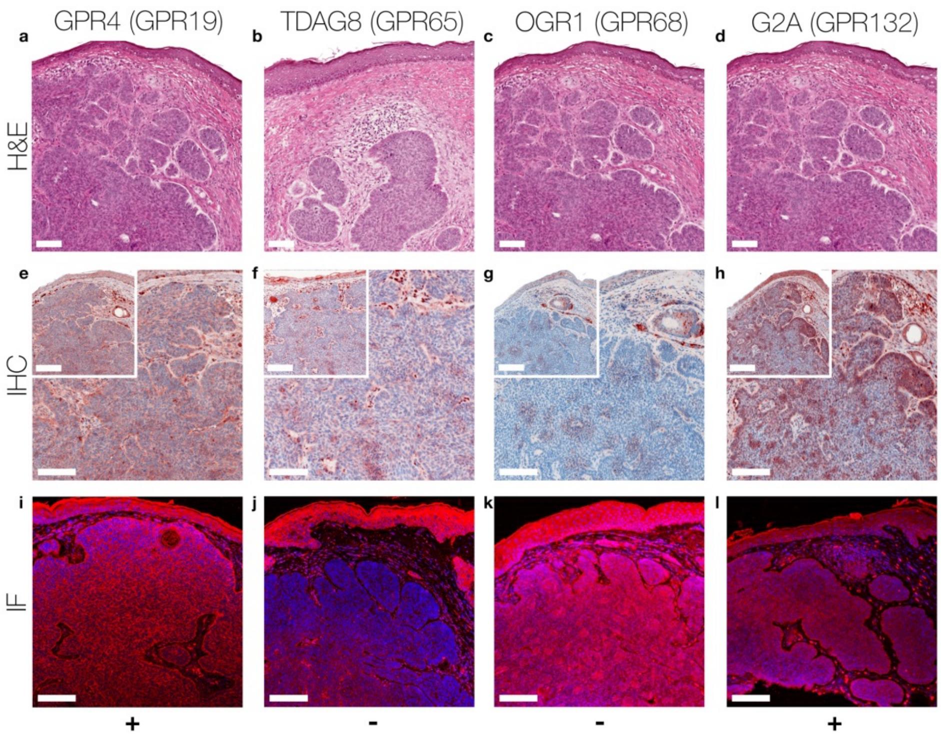

Figure S16: Immunohistochemistry and Immunofluorescence of BCC.

Immunohistochemical and immunofluorescent staining for GPR4 (GPR19), TDAG8 (GPR65), OGR1 (GPR68) and G2A (GPR132) in BCC tissue. (a-d) histochemical H&E staining, (e-h) immunohistochemical staining, inserted images present a 2x larger field of view, (i-l) immunofluorescence staining, red: secondary antibody label, blue: DAPI. Scale bars correspond to 100 μm (a-l: patient 24). Scores (bottom row) were assigned for ++: strong positive/positive reactions; +: weak positive/partial positive reaction; -: negative reaction. The expression of GPR4 and G2A is weak positive on BCC, even though the tumor cells of G2A in the periphery appear to be strong positive in comparison the tumor cells in the centre. There is no expression of TDAG8 and OGR1.

Figure S17

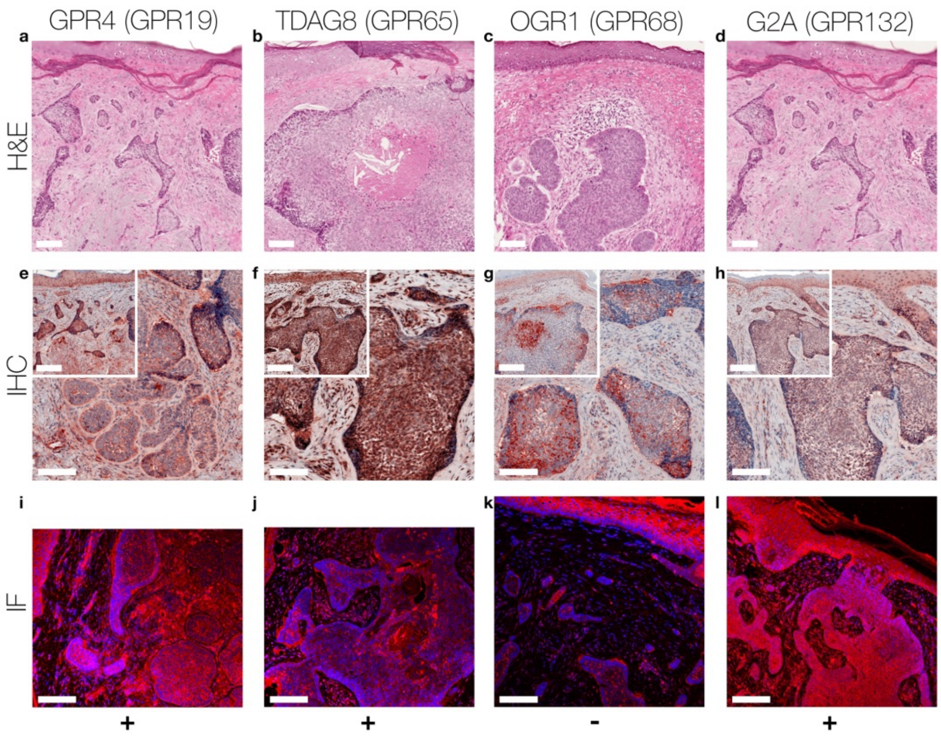

Figure S17: Immunohistochemistry and Immunofluorescence of BCC.
Immunohistochemical and immunofluorescent staining for GPR4 (GPR19), TDAG8 (GPR65), OGR1 (GPR68) and G2A (GPR132) in BCC tissue. (a-d) histochemical H&E staining, (e-h) immunohistochemical staining, inserted images present a 2x larger field of view, (i-l) immunofluorescence staining, red: secondary antibody label, blue: DAPI. Scale bars correspond to 100 μm (a-l: patient 25). Scores (bottom row) were assigned for ++: strong positive/positive reactions; +: weak positive/partial positive reaction; -: negative reaction. Except of the strong positive expressed BCC tumor cells in the periphery of GPR4 and TDAG8, GPR4 as well as TDAG8 and G2A are expressed partial positive. There is no expression of OGR1.

Figure S18

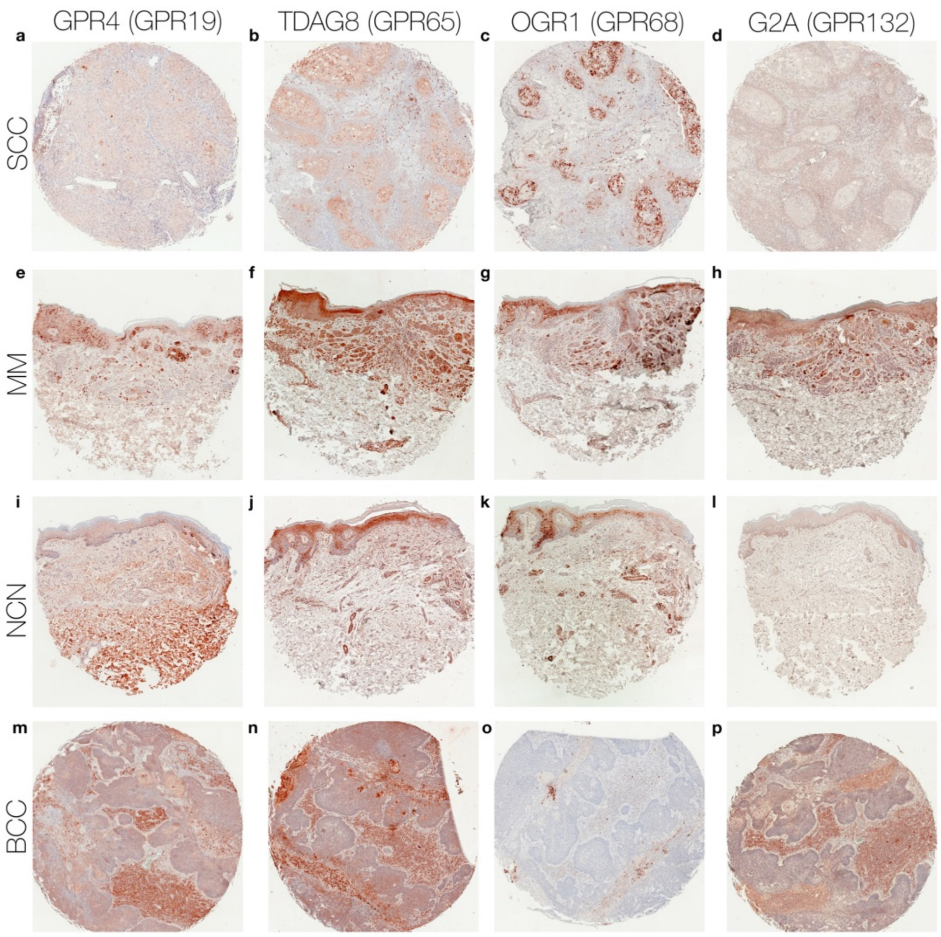

Figure S18: Tissue microarray of SCC, MM, NCN and BCC.
Selection of Immunohistochemical tissue microarray staining for GPR4 (GPR19), TDAG8 (GPR65), OGR1 (GPR68) and G2A (GPR132).

Supplementary tables

Table S1: Overview of general patient data. Overview of age and gender the stained tissue samples were taken from.

patient	gender	age
1	♀	86
2	♂	88
3	♀	85
4	♂	82
5	♀	87
6	♂	90
7	♀	81
8	♂	74
9	♂	34
10	♀	79
11	♀	87
12	♂	69
13	♂	59
14	♂	77
15	♀	68
16	♀	20
17	♂	30
18	♀	49
19	♂	51
20	♂	90
21	♂	90
22	♀	51
23	♂	45
24	♂	84
25	♂	74
26	♂	59
27	♂	80
28	♂	80
29	♂	84
30	♂	74
31	♀	94
32	♀	87
33	♂	73
34	♀	22
35	♀	89
36	♂	51
37	♂	48
38	♂	58
39	♀	66

40	♂	80
41	♂	69
42	♀	45
43	♀	93
44	♀	46
45	♂	69
46	♀	45
47	♀	45
48	♀	74
49	♂	83
50	♀	45
51	♂	83
52	♀	59
53	♂	68
54	♀	36
55	♂	53
56	♂	16
57	♂	55
58	♀	26
59	♀	48
60	♀	59
61	♀	69
62	♂	50
63	♀	69
64	♀	68
65	♂	50
66	♀	33
67	♂	28
68	♀	71
69	♀	27
70	♀	27
71	♀	28
72	♂	85
73	♀	37
74	♀	46
75	♂	69
76	♂	68
77	♂	66
78	♀	44
79	♂	88
80	♀	15
81	♀	50
82	♀	50
83	♀	19

84	♂	27
85	♀	19
86	♀	28
87	♀	43
88	♂	40
89	♂	64
90	♂	10
91	♂	42
92	♀	14
93	♂	38
94	♂	52
95	♀	44
96	♂	38
97	♀	43
98	♀	31
99	♂	11
100	♀	19
101	♀	30
102	♀	8
103	♂	54
104	♀	82
105	♂	77
106	♂	77
107	♂	65
108	♂	64
109	♂	78
110	♂	65
111	♀	65
112	♀	75
113	♀	85
114	♀	83
115	♂	66
116	♂	69
117	♂	98
118	♀	55
119	♀	65
120	♀	71
121	♂	59
122	♀	76
123	♂	69
124	♂	81
125	♂	78
126	♂	83
127	♀	84

Table S2: Scores of SCC. Scores of the staining for GPR4 (GPR19), TDAG8 (GPR65), OGR1 (GPR68) and G2A (GPR132) on SCC cells. ++: strong positive/positive reaction; +: weak positive/ partial positive reaction; -: negative reaction; N/A not available, TMA_{SCC} 1-26: patient 26-51.

TMA_{SCC}	GPR4	TDAG8	OGR1	G2A
1	N/A	N/A	N/A	N/A
2	+	+	+	+
3	+	+	+	+
4	+	+	-	+
5	+	+	-	+
6	+	+	-	+
7	-	+	-	+
8	+	N/A	N/A	-
9	+	+	+	+
10	+	+	-	+
11	+	++	-	-
12	+	+	+	+
13	+	++	++	N/A
14	+	+	+	+
15	+	+	-	+
16	+	+	++	+
17	+	+	+	+
18	++	+	+	++
19	+	+	++	++
20	+	+	N/A	+
21	+	++	+	+
22	+	+	-	+
23	+	N/A	+	+
24	+	+	+	+
25	+	+	-	+
26	+	+	-	+

Table S3: Scores of MM. Scores of the staining for GPR4 (GPR19), TDAG8 (GPR65), OGR1 (GPR68) and G2A (GPR132) on MM cells. ++: strong positive/positive reaction; +: weak positive/ partial positive reaction; -: negative reaction; +N/A not available, TMA_{MM} 1-27: patient 52-78.

TMA_{MM}	GPR4		TDAG8		OGR1		G2A	
	epidermal	dermal	epidermal	dermal	epidermal	dermal	epidermal	dermal
1	N/A	N/A	+ 3)	+ 2)	++	++	+ 3)	++
2	++	- 3)	+ 2)	++	N/A	N/A	++	++
3	++	++	++	++	++	++	++	++
4	++	++ 1)	++	-	++	-	++	+ 1) 3)
5	++	++	++	++	++	+ 2)	++	+ 3)
6	N/A	N/A	+	+	N/A	N/A	N/A	N/A
7	++	++ 1)	+ 3)	+ 2)	++	+ 2)	++	+ 1) 3)
8	++	++ 1)	++	++	++	++ 4)	+	+ 3)
9	++	- 3)	++	++	++	+ 2)	+	+ 3)
10	++	++ 1)	++	+ 2)	++	- 1)	+	+ 1) 3)
11	++	- 2)	++	++	++	++ 4)	+	+
12	N/A	N/A	N/A	N/A	N/A	N/A	+	N/A
13	++	- 2)	++	++	++	++	+	+
14	+	++	++	++	++	++	++	+ 3)
15	++	++	++	++	+	+	+	+ 3)
16	N/A	N/A	N/A	N/A	N/A	N/A	N/A	N/A
17	++	-	+	+	++	+	N/A	N/A
18	N/A	N/A	N/A	N/A	N/A	N/A	N/A	N/A
19	++	+ 2)	++	++	++	+	++	+ 3)
20	++	+ 2)	++	++	++	+ 2)	++	+ 3)
21	++	++ 1)	N/A	N/A	++	+	++	+ 3)
22	N/A	N/A	++	++	++	+	N/A	N/A
23	++	-	++	++	++	+ 2)	+	+
24	N/A	N/A	++	+	++	+ 2)	+	+
25	++	++ 1)	++	++	++	+	++	+ 1) 3)
26	++	+ 3)	++	++	++	+	++	+ 1) 3)
27	N/A	N/A	++	++	++	+	+	+ 3)

1) strong positive either on the surface or in the deeper parts of the tumor tissue

2) single tumor cells are strong positive, but the overall impression is weak positive expression

3) large tumor cells appear to be strong positive

4) partially strong positive

Table S4: Scores of NCN. Scores of the staining for GPR4 (GPR19), TDAG8 (GPR65), OGR1 (GPR68) and G2A (GPR132) on NCN cells. ++: strong positive/positive reaction; +: weak positive/ partial positive reaction; -: negative reaction; N/A not available, TMA_{NCN} 1-24: patient 79-102

TMA_{NCN}	GPR4		TDAG8		OGR1		G2A	
	epidermal	dermal	epidermal	dermal	epidermal	dermal	epidermal	dermal
1	+	+ 5)	N/A	+ 3)	-	+ 5)	-	+ 2) 5)
2	N/A	+ 5)	+	++	-	+	-	+ 5)
3	+	+ 3) 5)	N/A	N/A	-	+	+ 2)	+ 1) 3)
4	+	+ 3) 5)	N/A	++	-	+ 5)	-	+ 1) 3)
5	++	N/A	N/A	N/A	N/A	N/A	N/A	N/A
6	+	- 3)	+	++	-	+ 5)	+ 2)	+ 2) 5)
7	+	+ 3)	-	++	-	+ 5)	-	++ 4)
8	++	+ 5)	++	++	N/A	N/A	N/A	N/A
9	+	- 3)	+	+ 3)	+ 2)	+	++	- 3)
10	N/A	N/A	+	+	+ 2)	+	+ 2)	+
11	N/A	N/A	N/A	+ 3)	+ 2)	+	N/A	+ 1) 3)
12	N/A	N/A	+	+ 3)	N/A	N/A	-	+
13	+	+ 3)	+	N/A	N/A	N/A	N/A	N/A
14	++	+ 3)	++	++	N/A	N/A	++ 4)	+ 1) 3)
15	+	+ 5)	+	++	-	+	+	+
16	N/A	N/A	+	++	N/A	N/A	N/A	N/A
17	++	N/A	+	+	-	+ 5)	N/A	N/A
18	+	+ 5)	++	++	-	+	+	+ 5)
19	++	N/A	++	N/A	N/A	N/A	++ 4)	N/A
20	++	+ 3) 5)	+	+ 3)	+ 2)	+ 5)	++ 4)	+ 3) 5)
21	N/A	N/A	++	N/A	N/A	N/A	N/A	N/A
22	+	+ 3)	+ 3)	+ 3)	+ 2)	+ 2)	+ 5)	+ 3) 5)
23	++	+	N/A	++	N/A	N/A	N/A	+ 5)
24	+	+ 3) 5)	N/A	++	N/A	+	N/A	+ 3)

1) strong positive either on the surface or in the deeper parts of the tumor tissue

2) single tumor cells are strong positive, but the overall impression is weak positive expression

3) large tumor cells appear to be strong positive

4) partially strong positive

5) weak positive

Table S5: Scores of BCC. Scores of the staining for GPR4 (GPR19), TDAG8 (GPR65), OGR1 (GPR68) and G2A (GPR132) on BCC cells. ++: strong positive/positive reaction; +: weak positive/ partial positive reaction; -: negative reaction; N/A not available, TMA$_{BCC}$ 1-25: patient 103-127

TMA$_{BCC}$	GPR4	TDAG8	OGR1	G2A
1	+ [5]	+ [5]	-	++
2	N/A	N/A	N/A	N/A
3	+ [5]	+ [5]	+ [6]	+ [5]
4	-	+ [5]	+ [6]	+ [5]
5	N/A	+ [6]	-	N/A
6	+	N/A	+ [6]	+ [5]
7	+	+ [5]	+ [6]	N/A
8	+	++	+ [6]	++
9	+	+ [5]	-	+ [5]
10	++	+ [5]	-	++
11	+ [5]	+ [5]	-	+ [5]
12	++	++	+ [6]	+ [5]
13	+ [5]	+ [5]	+ [5]	+ [5]
14	++	N/A	+ [6]	++
15	++	+ [6]	-	+ [5]
16	++	++	+ [6]	+ [5]
17	+	++	+ [6]	N/A
18	++	++	+ [5]	++
19	+	++	+ [6]	+ [5]
20	++	+ [5]	+ [6]	++
21	++	++	+ [6]	++
22	++	++	+	+ [5]
23	++	++	+ [6]	+ [5]
24	++	+ [5]	+ [6]	++
25	+ [5]	+ [5]	+ [6]	N/A

[5] weak positive

[6] partial positive

Figure S19

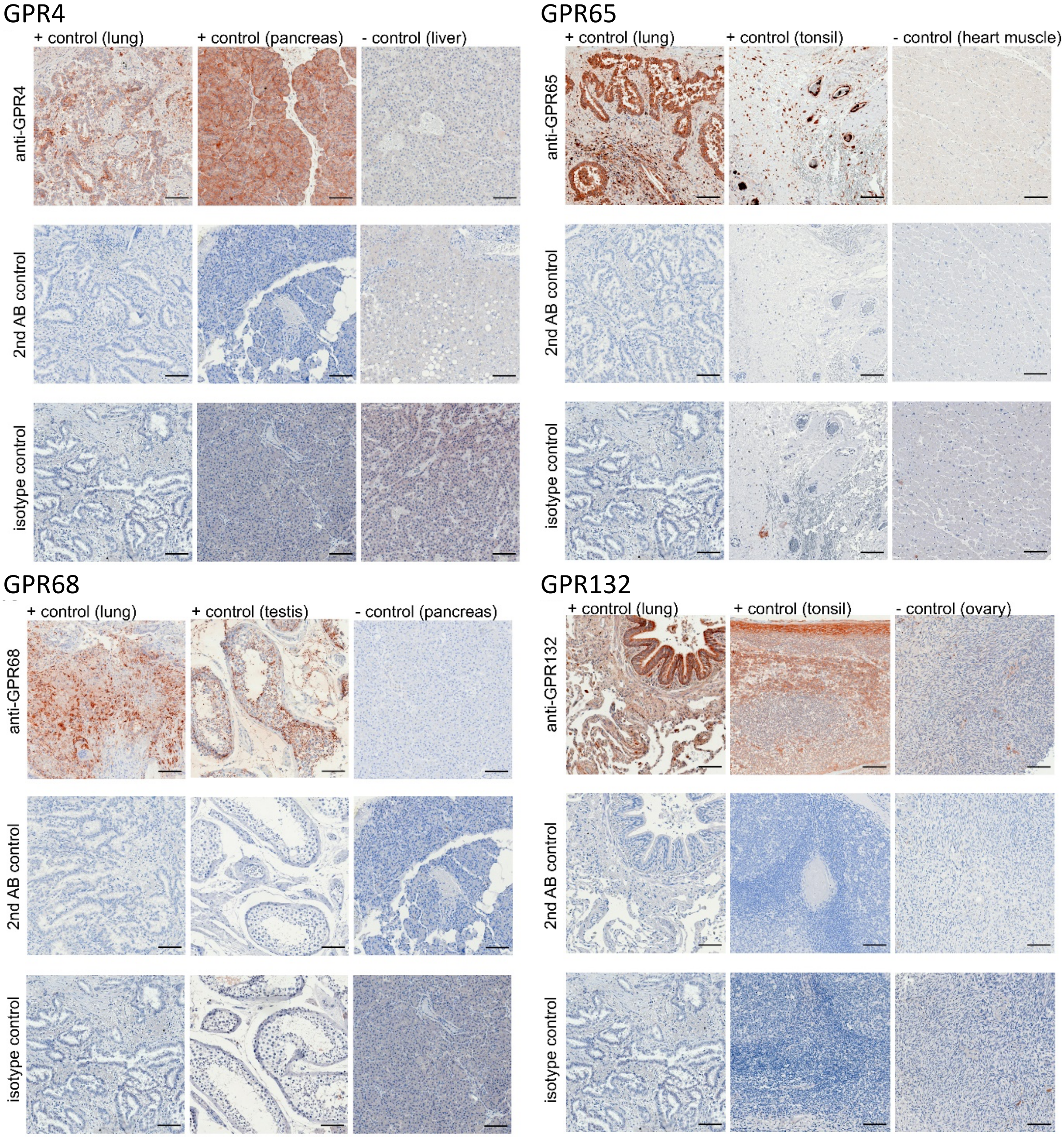

Figure S19: Tissue controls for IHC/TMA staining with anti-pH-GPCR antibodies.
IHC staining on positive and negative control tissue. One positive control tissue was each selected according to the primary antibody datasheet and a second positive control and a negative control for each pH-GPCR were selected according to the proteomics database "The Human Protein Atlas" (www.proteinatlas.org). Control tissue were stained with the respective anti pH-GPCR antibody, the isotype control antibody and without primary antibody (secondary antibody control: 2nd AB control). Scale bars correspond to 100 µm.

Figure S20

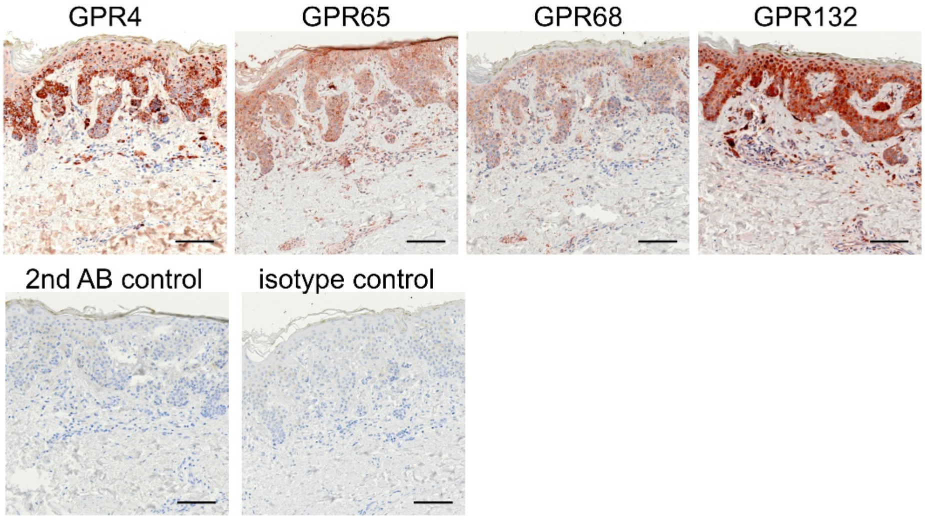

Figure S20: Skin tumor tissue control for IHC/TMA staining with anti-pH-GPCR antibodies. IHC staining on a malignant melanoma tissue section with the four anti-pH-GPCR antibodies, the isotype control antibody and without primary antibody (secondary antibody control: 2nd AB control). Scale bars correspond to 100 µm.
